# Timely binding of IHF and Fis to *DARS2* regulates ATP–DnaA production and replication initiation

**DOI:** 10.1093/nar/gku1051

**Published:** 2014-11-06

**Authors:** Kazutoshi Kasho, Kazuyuki Fujimitsu, Toshihiro Matoba, Taku Oshima, Tsutomu Katayama

**Affiliations:** 1 Department of Molecular Biology, Graduate School of Pharmaceutical Sciences, Kyushu University, Fukuoka 812-8582, Japan; 2 Division of Genomics of Bacterial Cell Functions, Graduate School of Biological Sciences, Nara Institute of Science and Technology, Ikoma, Nara 630-0192, Japan

## Abstract

In *Escherichia coli*, the ATP-bound form of DnaA (ATP–DnaA) promotes replication initiation. During replication, the bound ATP is hydrolyzed to ADP to yield the ADP-bound form (ADP–DnaA), which is inactive for initiation. The chromosomal site *DARS2* facilitates the regeneration of ATP–DnaA by catalyzing nucleotide exchange between free ATP and ADP bound to DnaA. However, the regulatory mechanisms governing this exchange reaction are unclear. Here, using *in vitro* reconstituted experiments, we show that two nucleoid-associated proteins, IHF and Fis, bind site-specifically to *DARS2* to activate coordinately the exchange reaction. The regenerated ATP–DnaA was fully active in replication initiation and underwent DnaA–ATP hydrolysis. ADP–DnaA formed heteromultimeric complexes with IHF and Fis on *DARS2*, and underwent nucleotide dissociation more efficiently than ATP–DnaA. Consistently, mutant analyses demonstrated that specific binding of IHF and Fis to *DARS2* stimulates the formation of ATP–DnaA production, thereby promoting timely initiation. Moreover, we show that IHF–*DARS2* binding is temporally regulated during the cell cycle, whereas Fis only binds to *DARS2* in exponentially growing cells. These results elucidate the regulation of ATP–DnaA and replication initiation in coordination with the cell cycle and growth phase.

## INTRODUCTION

**Figure 1.**
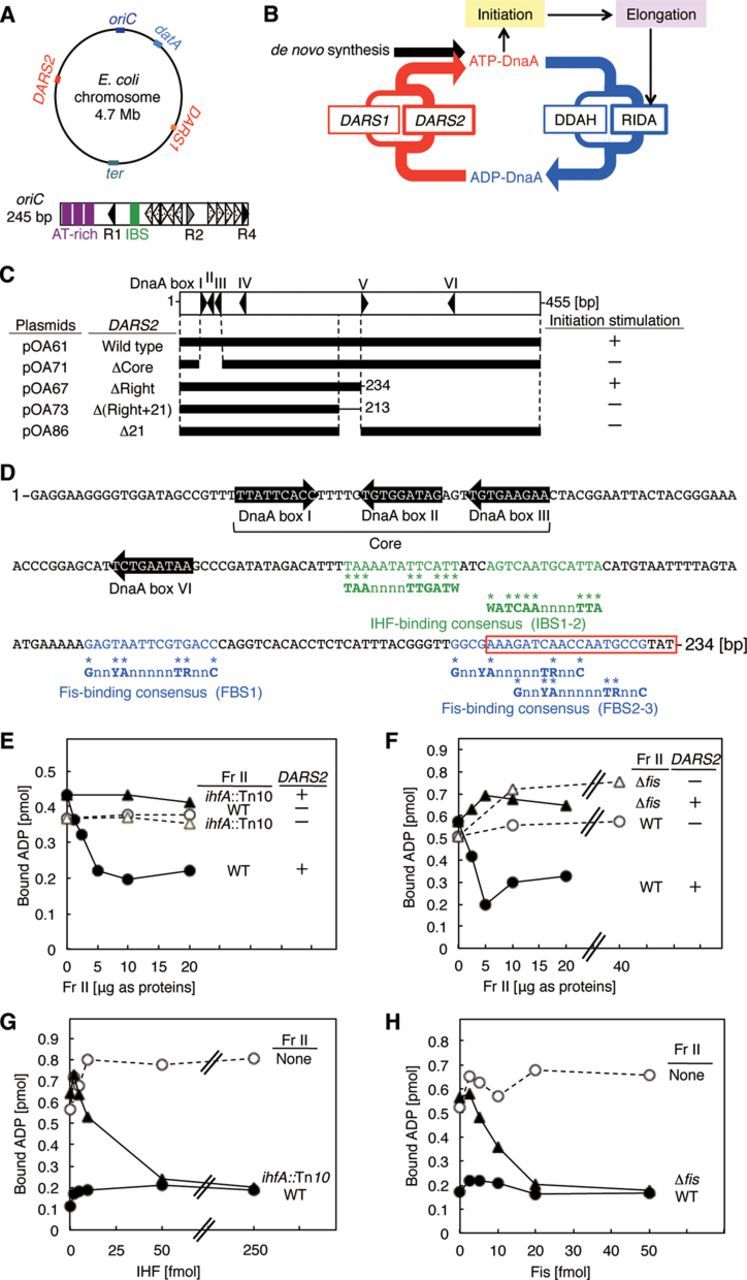
A minimal *DARS2* and roles for IHF and Fis. (**A**) Overall structure of the *E. coli* chromosome and *oriC*. The upper figure indicates the relevant loci on the circular chromosome. The lower figure indicates the basic structure of *oriC* (open rectangle). Filled, gray and dotted arrowheads indicate the high affinity DnaA boxes (R1 and R4), moderate affinity site (R2) and low affinity sites, respectively. The three AT-rich repeats in the duplex unwinding element (AT-rich) and the IBS are also shown by purple bars and a green bar, respectively. (**B**) Schematic presentation of the regulatory cycle of DnaA. ATP–DnaA initiates replication, resulting in activation of replisomes and RIDA. In addition, DDAH is activated by timely binding of IHF to *datA*. *DARS1* and *DARS2* regenerate ATP–DnaA from ADP–DnaA; *invivo*, *DARS2* plays the predominant role in this process. (**C**) *DARS2* deletion analysis. The open rectangle indicates the whole *DARS2* region. Filled bars indicate regions carried on vector pACYC177. Cells bearing these plasmids were grown at 37°C in supplemented M9-ampicillin medium, and then subjected to flow cytometry analysis (see Supplementary Figure S1A for data). +, active in stimulation of initiation; -, inactive. (**D**) Sequence of the minimal *DARS2* region on pOA67. Consensus binding sequences of IHF (green letters) and Fis (blue letters) are TAAnnnnTTGATW (where W is A or T) and GnnYAnnnnnTRnnC (Y = C or T; R = A or G; n is any nucleotide) (6,19), respectively. The red box represents the critical 21-mer. (**E**) *DARS2* reaction using IHF-defective crude protein extracts. [^3^H]ADP–DnaA (2 pmol) and 5 fmol of pOA61 (*DARS2*, +) or pACYC177 (*DARS2*, −) were co-incubated at 30°C for 15 min with a crude protein extract (Fr II) of YH014 (WT) or YH014-I (*ihfA*::Tn*10).* See also Supplementary Figure S2A. (**F**) *DARS2* reaction using Fis-defective crude protein extracts. Similar experiments were performed using a crude protein extract of MG1655 (WT) or KMG-2 (Δ*fis*). See also Supplementary Figure S2B. (**G**) *In vitro* complementation experiments for IHF. Similar experiments were performed using pOA61 and purified IHF with no extract (None) or with a crude extract (Fr II, 5 μg) from YH014 (WT) or YH014-I (*ihfA*::Tn*10).*(H) *In vitro* complementation experiments for Fis. Similar experiments were performed using pOA61 and purified Fis with no extract (None) or crude extract (Fr II, 5 μg) from MG1655 (WT) or KMG-2 (Δ*fis*).

Initiation of chromosomal DNA replication is rigidly regulated to occur only once, at the appropriate time, during each cell cycle. In *Escherichia coli*, the proteins DnaA and IHF play crucial roles in initiating replication at the chromosomal origin, *oriC* (1–4) (Figure 1A). DnaA, a member of the AAA+ ATPase family, has an exceptionally high affinity for both ATP and ADP, but only ATP–DnaA is active in initiation. IHF, a member of the nucleoid-associated proteins family, sharply bends DNA at the IHF-binding site (IBS) (5,6). *oriC* contains an AT-rich duplex unwinding element, a single IBS and at least 12 specific DnaA-binding sites (DnaA boxes) with various affinities (Figure 1A) (3,7,8). Binding of IHF and ATP–DnaA molecules to *oriC* induces a conformational change in the DNA at the origin, leading to unwinding of the duplex unwinding element (1,3,9). This step is followed by successive loading of DnaB helicase, DnaG primase and DNA polymerase III holoenzyme, which perform DNA synthesis (10). ADP–DnaA can also form multimers on *oriC*, but these complexes are inactive in initiation.

The cellular level of ATP–DnaA is tightly regulated by negative and positive regulatory pathways and peaks at the time of replication initiation (11). During replication, DnaA-bound ATP is hydrolyzed by a complex containing Hda and the DNA-loaded clamp subunit of DNA polymerase III holoenzyme, yielding initiation-inactive ADP–DnaA. This replication-coupled negative feedback on DnaA is called RIDA (regulatory inactivation of DnaA; Figure 1A) (2,12). Cells defective in RIDA exhibit constitutively elevated levels of ATP–DnaA and excess initiations, resulting in inhibition of colony formation (12,13). In addition, the chromosomal locus *datA*, which contains a cluster of DnaA boxes and an IBS, is required to prevent initiation from occurring at inappropriate times (14–16). In a recent study, we showed that ATP–DnaA molecules form oligomers specifically on IHF-bound *datA*, and that this oligomerization stimulates DnaA–ATP hydrolysis (17). IHF binding to *datA* is specific to the post-initiation stage of the replication cycle (17). This system for timely inactivation of DnaA, termed DDAH (*datA*-dependent DnaA–ATP hydrolysis), supports RIDA (Figure 1B). In addition, *dnaA* gene transcription is autoregulated; it is inhibited more effectively by ATP–DnaA than by ADP–DnaA (16). This inhibition represses *de novo* ATP–DnaA synthesis around the time of initiation, indirectly assisting RIDA and DDAH.

In contrast to RIDA and DDAH, *DARS*s (DnaA-reactivating sequences) increase the level of ATP–DnaA by promoting nucleotide exchange on ADP–DnaA (Figure 1B) (18). The *E. coli* chromosome contains at least two *DARS*s, *DARS1* and *DARS2*, which are required for timely initiation of replication during the cell cycle. Structurally, these sites share a highly conserved core region containing three DnaA boxes that are necessary for nucleotide exchange (Figure 1C and D). ADP–DnaA oligomers formed on *DARS1* promote dissociation of ADP via specific interactions between DnaA molecules, leading to a release of apo-DnaA from the complexes. These apo-DnaA molecules then bind ATP, resulting in the regeneration of ATP–DnaA. In contrast to *DARS1*, the *in vitro* activity of *DARS2* requires a crude protein extract, suggesting that unidentified proteins regulate *DARS2* activity. In addition, *DARS2* increases the cellular ATP–DnaA level and stimulates initiation more effectively than *DARS1*, suggesting that activators for *DARS2* play critical roles in regulation of the ATP–DnaA level and initiation (18). However, the nature of the *DARS2* activators and how *DARS2* function is regulated during the cell cycle or under different growth conditions remains unknown.

In this study, we identified two nucleoid-associated proteins, IHF and Fis, as key *DARS2* activators. Fis binds to DNA in a site-specific manner and regulates gene expression, recombination and superhelicity (5). Fis also stimulates replication initiation, but the underlying mechanism remains unknown (19,20,21). We reconstituted *DARS2*-dependent DnaA reactivation *in vitro* using purified IHF and Fis. Simultaneous binding of IHF and Fis to specific sites within *DARS2* promoted regeneration of ATP–DnaA. Cell-cycle analyses revealed that IHF bound to *DARS2* in a temporally regulated manner. By contrast, Fis bound to *DARS2* specifically during the exponential growth phase in a cell cycle–independent manner. Based on these observations, we propose that the activation of *DARS2* is regulated by two pathways: an IHF pathway that coordinates activation with the cell cycle, and a Fis pathway that coordinates activation with growth phase. The requirement for the simultaneous binding of IHF and Fis to *DARS2* for ATP–DnaA regeneration would ensure the proper timing of chromosomal replication.

## MATERIALS AND METHODS

### Strains, DNA and proteins

Strains, DNA and proteins used in this study are all described in Supplementary Data.

### Reconstitution of *DARS2* reactions and DnaA cycle *in vitro*

*DARS2*–mediated ADP dissociation was performed with a crude protein extract (fraction [Fr] II) and poly (dI-dC) as described previously (18). Reconstituted *DARS2* reactions containing IHF and Fis were performed under similar buffer conditions (for details, see Supplementary Data). *DARS2*–mediated ATP–DnaA regeneration was reconstituted under the same conditions used for ADP dissociation, except that 1.5 μM [α-^32^P] ATP was added. In the DnaA cycle reconstitution system integrating RIDA or DDAH, regenerated ATP–DnaA was incubated with IHF–*datA* or DNA–clamp–Hda complexes, respectively, as described previously (17,18). *oriC* replication coupled with *DARS2*–mediated ATP–DnaA regeneration was performed using purified replication proteins as described previously (22), except that the reactions contained 80 nM IHF instead of HU, 5 fmol pOA61 or pACYC177, the indicated amounts of Fis and 80 nM DnaG. The details of these reactions are also provided in Supplementary Materials and Methods.

### Electrophoretic mobility shift assay

Proteins were incubated at 30°C for 5 min in buffer GS (20 mM Hepes-KOH [pH 7.6], 50 mM potassium glutamate, 10 mM magnesium acetate, 1 mM ethylenediaminetetraacetic acid [EDTA], 8 mM dithiothreitol, 100 μg/ml bovine serum albumin and 5% glycerol) or dissociation buffer (20 mM Tris–HCl [pH 7.5], 100 mM potassium glutamate, 10 mM magnesium acetate, 2 mM ATP, 8 mM dithiothreitol and 100 μg/ml albumin) containing the indicated DNAs and poly (dI-dC), and then subjected to polyacrylamide gel electrophoresis (PAGE) in Tris-borate buffer and stained with GelStar. For details, see Supplementary Materials and Methods.

### DNase I footprint analysis

The upper or lower strand of *DARS2* was labeled by polymerase chain reaction (PCR) using ^32^P-labeled primers. The labeled *DARS2* DNA (100 fmol) was incubated at 30°C for 10 min in buffer GS (10 μl) containing indicated amounts of IHF or Fis, 120 ng poly (dI-dC), 5 mM calcium acetate and 12.5 miliunits of DNase I (New England Biolabs) and then subjected to 7 M urea-5% PAGE and phosphorimaging as described (7,21).

### Pull-down assay and chromatin immunoprecipitaion

Pull-down experiments and chromatin immunoprecipitaion (ChIP) were performed essentially as described previously (17,23). For details, see Supplementary Materials and Methods.

### Determination of *oriC* copy number and the cellular ATP–DnaA level

The experiments were done, as described (18). The details of these experiments are also provided in Supplementary Materials and Methods.

## RESULTS

### Definition of a minimal *DARS2* region

*DARS1* and *DARS2* are required for timely initiation of replication (18) and contain the core DnaA boxes that promote the formation of DnaA oligomers, a crucial step in mediating ADP dissociation and regenerating ATP–DnaA from ADP–DnaA. The resulting increase of the ATP–DnaA level is a prerequisite for timely initiation *in vivo*. *DARS2* rather than *DARS1* plays the dominant factor in mediating ATP–DnaA regeneration *in vivo*. In addition, unlike *DARS1*, *DARS2* requires *trans*-acting activators for ADP dissociation (18). It is plausible that the overall conformation of the *DARS2*-DnaA complex is affected by activators that promote specific inter-DnaA interactions within the DnaA-oligomer, leading to structural changes that reduce the affinity of DnaA for ADP. As previously suggested for *DARS1* (18), apo-DnaA may be released from the *DARS2*-DnaA complex and then bind to ATP, thereby regenerating ATP–DnaA. Also, activators for *DARS2* could play a crucial role in the timely regulation of *DARS2* function in the cell cycle and during cell growth phase. In this study, we searched for activating factors of *DARS2*.

Based on the assumption that *DARS2* activators directly bind *DARS2*, we first defined the minimal region of *DARS2* necessary for stimulation of initiation, reasoning that this region should contain specific binding sequences for activators. To this end, we ligated three truncated *DARS2* derivatives, ΔRight, ΔRight+21 and Δ21, to the moderate–copy number plasmid pACYC177 (Figure 1C and D), and then performed flow cytometry analysis on cells bearing the resultant plasmids. To determine the number of *oriC* copies, growing cells were incubated in the presence of rifampicin and cephalexin (inhibitors of initiation and cell division, respectively) until the entire chromosome was replicated. After this procedure, the number of chromosomes per cell, deduced by flow cytometry, corresponds to the number of *oriC* copies per cell (16). In rapidly growing cells, initiation occurs while the previous round of replication is ongoing, resulting in populations of cells containing predominantly two or four (or four or eight) copies of *oriC*.MG1655 cells bearing pACYC177 predominantly contain two or four copies of *oriC* (Supplementary Figure S1A). Introduction of a plasmid containing wild-type *DARS2* [pOA61, pACYC177-*DARS2*], but not a mutant lacking the DnaA-box cluster [pOA71, ΔCore], stimulated overinitiation of replication, which was reflected by the increase in *oriC* copy number (Figure 1B and Supplementary Figure S1A), as previously demonstrated (18). The slightly increased size (mass) of cells bearing pOA61 has been attributed to retardation of cell division by overinitiation (24). Introduction of pOA67 [ΔRight], which lacks the right half of *DARS2*, also caused overinitiation, whereas introduction of pOA73 [Δ(Right+21)] or pOA86 [Δ21] did not (Figure 1C and Supplementary Figure S1A). Because the latter two plasmids have a 21-bp deletion flanking the right-half region, these results indicate that *DARS2* ΔRight contains the minimal *DARS2*, and that the 21-mer sequence is crucial for *DARS2* activation (Figure 1D). Consistent with this, DnaA boxes IV–VI in *DARS2* were dispensable for stimulation of initiation under these experimental conditions (Supplementary Figure S1B).

### IHF and Fis stimulate *DARS2*-mediated ADP dissociation

Sequence analysis revealed that the minimal *DARS2* region contains two 13-mer IHF-binding consensus sequences (IBS1–2) and three 15-mer Fis-binding consensus sequences (FBS1–3) (Figure 1D). The crucial 21-mer sequence contains FBS2–3. IHF is a heterodimer of α and β subunits, and Fis is a homodimer (5). To investigate the roles of IHF and Fis in *DARS2* activation, we assessed ADP dissociation from DnaA using a crude protein extract prepared from wild-type cells or cells defective in *ihfA* (encoding the IHF α-subunit) or *fis* (encoding Fis). A wild-type cell extract (fraction [Fr] II) promoted ADP dissociation from DnaA in a *DARS2*-dependent manner, as reported previously (18), but an extract prepared from either mutant did not (Figure 1E and F). Even low levels of IHF and Fis restored ADP dissociation to the wild-type level in the corresponding mutant extracts (Figure 1G and H). Western blotting analysis indicated that 5 μg of wild-type Fr II contained about 330 fmol IHF and about 35 fmol Fis (Supplementary Figure S2A and B). This means that the level of Fis was the limiting factor determining the activity of wild-type Fr II (Figure 1E and F). In addition, these results suggest that Fis activity in wild-type Fr II (Figure 1F) and purified Fis activity used in the *in vitro* complementation experiments (Figure 1H) were similar. We previously reported that *DARS2* activators are resistant to heat and RNaseA (18), which is consistent with the high stabilities of IHF and Fis (5).

### *In vitro* reconstitution of *DARS2* reactions by IHF and Fis

**Figure 2.**
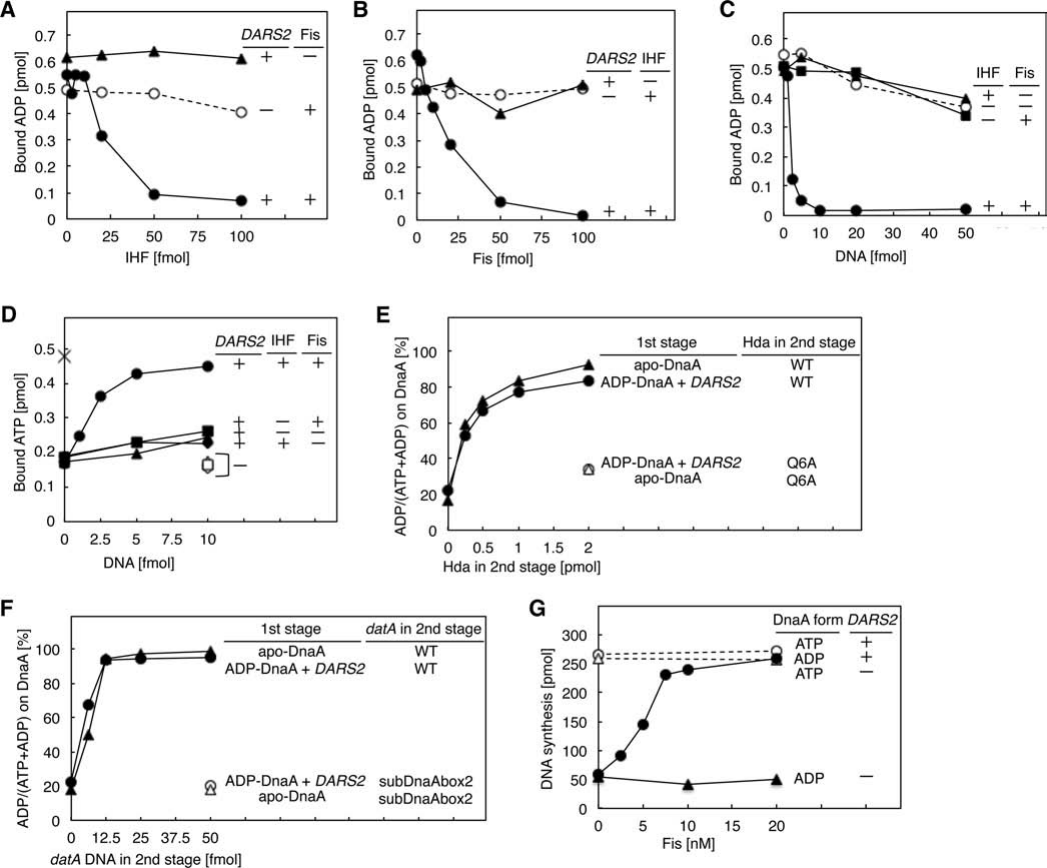
*In vitro* reconstitution of *DARS2* reactions. (**A**) ADP dissociation from DnaA. [^3^H]ADP–DnaA (2 pmol), the indicated amounts of IHF and 5 fmol of pOA61 (•,▴) or pACYC177 (○) were co-incubated at 30°C for 15 min without (▴) or with 50 fmol of Fis (•,○). See also Supplementary Figure S2C and D. (**B**) ADP dissociation from DnaA. Similar experiments were performed using the indicated amounts of Fis without (▴) or with 50 fmol of IHF (•,○). (**C**) ADP dissociation from DnaA. Similar experiments were performed using the indicated amounts of pOA61 without (○) or with 50 fmol each of Fis (•,▪) and IHF (•,▴). (**D**) ATP–DnaA generation. ADP–DnaA (2 pmol), 1.5 μM [α-^32^P]ATP and the indicated amounts of pOA61 (•,▪,▴,♦) or pACYC177 (○,□,Δ,⋄) were co-incubated at 30°C for 15 min with or without (▴,Δ) 50 fmol each of IHF (•,♦,○,⋄) and Fis (•,▪,○,□). apo-DnaA (×) was incubated similarly with [^32^P]ATP, but without DNA, IHF and Fis. (**E**) Reconstitution of the DnaA cycle using *DARS2* and RIDA. In the 1st stage, ADP–DnaA (•,○) or apo-DnaA (▴,Δ) was incubated as above with or without pOA61 (5 fmol) (*DARS2*) in buffer containing 1.5 μM [α-^32^P]ATP and 50 fmol each of IHF and Fis, followed by digestion of pOA61 with *Pci*I. In the 2nd stage, portions of the resultant reactions were incubated at 30°C for 20 min with the DNA-loaded clamp (40 fmol as the clamp) and wild-type Hda (WT) (•,▴) or the Hda Q6A mutant as a negative control (○,Δ). The Hda Q6A mutant is defective in clamp binding and RIDA (26). (**F**) Reconstitution of the DnaA cycle using *DARS2* and DDAH. Similar experiments were performed for the 1st stage. In the 2nd stage, the reactions were incubated at 30°C for 10 min with IHF (0.2 pmol) and 991-bp *datA* DNA (WT) (•,▴) or *datA* subDnaAbox2 mutant as a negative control (○,Δ). The *datA* DnaA box 2 is a crucial DnaA box for DDAH and the *datA* subDnaAbox2 is a mutant *datA* bearing a substituted DnaA box 2 sequence that is inactive in DDAH (17). (**G**) *oriC* replication coupled with *DARS2* ATP–DnaA regeneration. ADP–DnaA (•,▴) or ATP–DnaA (○,Δ) (0.5 pmol [20 nM] each) was incubated at 30°C for 15 min with 5 fmol of pOA61 (•,○) or pACYC177 (▴,Δ) in buffer containing M13KEW101 *oriC* plasmid (600 pmol nucleotides) and replication proteins, including 2 pmol (80 nM) of IHF and the indicated amounts of Fis.

We next reconstituted *DARS2*–mediated ADP dissociation from DnaA in reactions containing only IHF, Fis and the wild-type *DARS2* construct pOA61 (Figure 2A–C). The activities of IHF and Fis in this reconstituted reaction were similar to those used for the *in vitro* complementation of the IHF-defective and Fis-defective crude extracts (Figure 1G and H). By contrast, the activity of HU, a major nucleoid-associated protein and a structural homolog of IHF (5), was low in the ADP dissociation reaction (Supplementary Figure S2C and D), whereas HU is interconvertible with IHF in an *in vitro* reconstituted system for *oriC* plasmid replication (25).

Incubation of ADP–DnaA with *DARS2* and ATP yielded ATP–DnaA (Figure 2D). The ATP–DnaA generated was inactivated by RIDA or DDAH (Figure 2E and F), indicating full reconstitution of ATP–DnaA formation and the *DARS2*-RIDA/DDAH cycle *in vitro* (17,18,26). In addition, we successfully reconstituted *oriC* plasmid replication coupled with *DARS2* ATP–DnaA regeneration by IHF and Fis (Figure 2G). In these experiments, we used low concentrations of Fis that did not inhibit *oriC* replication in previously reported reconstituted systems (27).

Thus, the ATP binding, ATP hydrolysis and *oriC* initiation activities of DnaA were fully preserved in the reconstituted *DARS2*–mediated ADP dissociation reaction. Furthermore, these results support the idea that IHF and Fis are crucial activators of *DARS2.*

### Determination of IHF- and Fis-binding sites in *DARS2*

**Figure 3.**
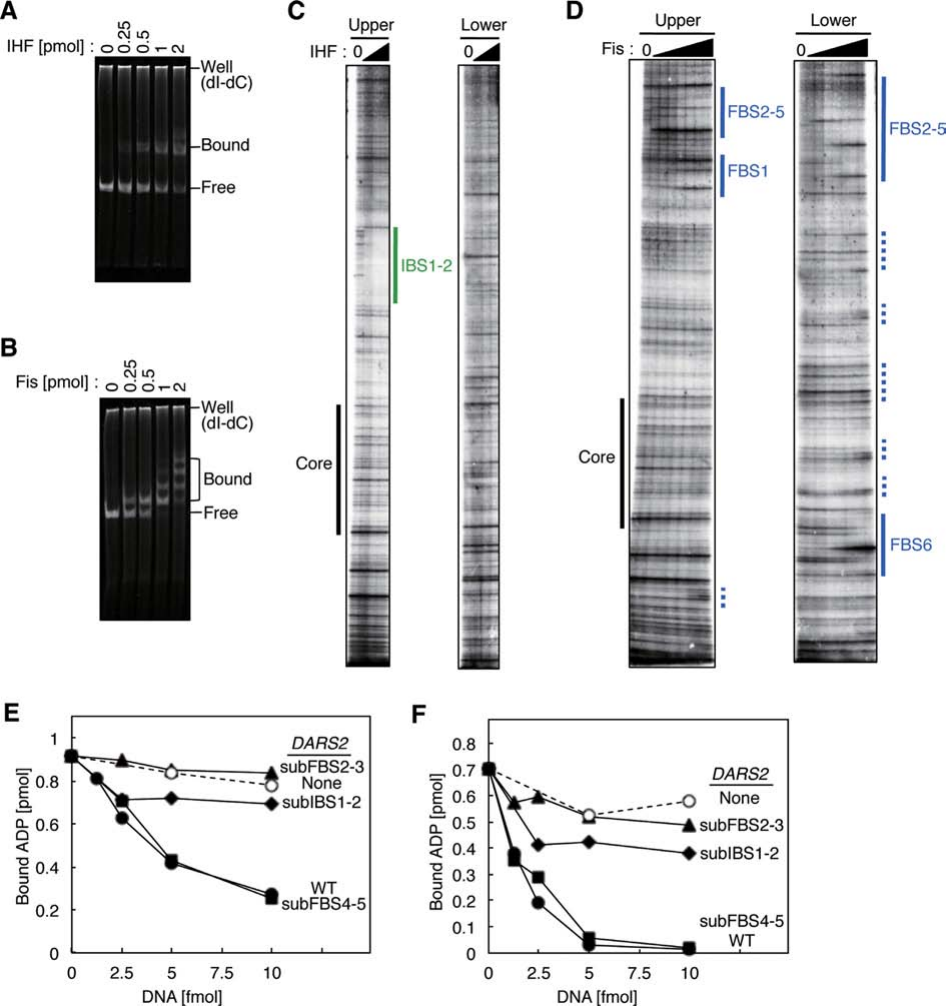
*DARS2* IBS1–2 and FBS2–3 are required for *DARS2* activation *in vitro*. (**A**, **B**) Electrophoretic mobility shift assay. IHF (A) or Fis (B) was incubated for 5 min at 30°C in buffer GS containing *DARS2* DNA (455 bp, 100 fmol) and poly (dI-dC) (120 ng), followed by 4% PAGE. Well; gel well, Bound; protein-bound DNA, Free; protein-free DNA. Poly (dI-dC) remained in the gel well. (**C**, **D**) Footprint analysis. The upper or lower strand of [^32^P]-*DARS2* (100 fmol) was incubated at 30°C for 10 min with DNase I and IHF (0, 1.2, 2.4, or 4.8 pmol) (C) or Fis (0, 0.12, 0.24, 0.48, 0.96, 1.9, 3.8, or 7.6 pmol) (D), followed by urea–PAGE and phosphorimaging. Green lines: IBS1–2. Blue lines: FBS1 FBS2–5, and FBS6. Blue dotted lines: low affinity Fis-binding sites. For detailed sequences, see also Supplementary Figure S3. (**E**, **F**) Roles for *DARS2* IBS and FBS in ADP dissociation from DnaA. MG1655 Fr II (5 μg) (E) or 50 fmol each of IHF and Fis (F) was co-incubated at 30°C for 15 min with [^3^H]ADP–DnaA (2 pmol) in the presence of pOA61 (WT) (•), pKX35 (subIBS1–2) (▴), pKX36 (subFBS2–3) (♦), pKX37 (subFBS4–5) (▪) or pACYC177 (None) (○). See also Supplementary Figures S4 and S5.

Next, to analyze the mechanism of *DARS2*–mediated ADP dissociation from DnaA, we determined the binding sites of IHF and Fis within *DARS2* using the electrophoretic mobility shift assay and DNase I footprinting (Figure 3A–D). The results of the electrophoretic mobility shift assay revealed that IHF and Fis directly bind to full-length *DARS2* (455 bp) with IHF binding to a predominant single site and Fis binding to at least four sites (Figure 3A and B).

In the DNase I footprint experiments, we used the upper and lower strands of *DARS2* to analyze the left and right halves of *DARS2*, respectively (Figure 3C and D). IHF protected a single region corresponding to IBS1–2 (Figure 3C and Supplementary Figure S3AB). Two IHF molecules might simultaneously bind to IBS1–2. Alternatively, IHF binding to these two sites might be mutually exclusive, and a mixture comprising *DARS2* molecules with IHF bound to IBS1 or to IBS2 might result in protection of the region including IBS1–2. IBS1 and IBS2 are separated by only 3 bp (Figure 1D and Supplementary Figure S3B). The close proximity of these sites may explain why IHF predominantly binds to a single site. DNA-bound IHF covers a 34-bp region, including the 13-bp IBS, and binding is accompanied by sharp DNA bending (6). IHF bound to IBS1 may become a physical obstacle inhibiting binding of IHF to IBS2, and *vice versa*. In addition, the large structural change induced by IHF binding might affect binding of the second IHF at the flanking site and the DNase I sensitivity of the IBS-flanking regions.

In addition, DNase I footprinting revealed that Fis protected three specific sites, FBS1, FBS2–5 and FBS6 (Figure 3D and Supplementary Figure S3AC). The FBS2–5 region includes four overlapping binding consensuses, but binding occurred predominantly on FBS2–3 (Figure 3D and Supplementary Figure S3C), consistent with our definition of the minimal *DARS2* sequence (Figure 1C and D). Several additional binding sites were also detected outside the minimal region when higher concentrations of Fis were present (Figure 3D), consistent with the weak affinity of Fis for AT-rich sites (28).

To confirm the specificity of the identified binding sites, we replaced them with scrambled sequences (Supplementary Figure S4A) and analyzed the resultant DNAs by the electrophoretic mobility shift assay. None of the substituted fragments bound IHF or Fis (Supplementary Figure S4B–F).

### IBS1–2 and FBS2–3 are required for ADP dissociation *in vitro*

To elucidate the functions of IBS and FBS, we assessed ADP dissociation from DnaA *in vitro* using *DARS2* derivatives bearing a substituted IBS or FBS (Supplementary Figure S4A). *DARS2* derivatives bearing substituted IBS1–2 and FBS2–3 (subIBS1–2 and subFBS2–3) were inactive for ADP dissociation in the presence of a crude protein extract (Figure 3E), whereas *DARS2* variants bearing subFBS1, subFBS4–5 or subFBS6 were fully active (Figure 3E and Supplementary Figure S5A). Similar results were obtained using an *in vitro* reconstituted system using purified IHF and Fis (Figure 3F and Supplementary Figure S5B). Thus, IBS1–2 and FBS2–3 are required for *DARS2* activation *in vitro*.

### Location of IBS1–2 and EBS2–3 is important in *DARS2* activation

IHF and Fis bend DNA by ∼180 and ∼60°, respectively (6,34). To assess the importance of DNA bending by IHF and Fis in *DARS2* activation, we analyzed *DARS2* derivatives in which the location of IBS1–2 or FBS2–3 was changed by 5 or 10 bp (Supplementary Figure S6A). A 5-bp translocation, but not a 10-bp translocation, would result in a change in the direction of the DNA bending. The activities of the *DARS2* derivatives were analyzed using the *in vitro* reconstituted system (Supplementary Figure S6B).

First, IBS1–2 was translocated by 5- or 10-bp toward FBS2–3, resulting in traIBS-R5 and –R10 (Supplementary Figure S6A). Compared with wild-type *DARS2*, *DARS2* traIBS-R5 was only partially effective in ADP dissociation, whereas *DARS2* traIBS-R10 was moderately effective (Supplementary Figure S6B). These results are consistent with the idea that IHF-dependent DNA bending is important for *DARS2* activation. The moderate activity of *DARS2* traIBS-R10 suggests that the distance between the core DnaA boxes and IBS1–2 is also important for *DARS2* function.

Next, the location of IBS1–2 was translocated by 5-bp or 10-bp toward the core DnaA boxes, resulting in traIBS-L5 and –L10 (Supplementary Figure S6A). Both of the *DARS2* mutants were only partially effective in ADP dissociation compared with the wild-type *DARS2* (Supplementary Figure S6B). This is consistent with the idea that the introduction of a shorter region between the core DnaA boxes and IBS1–2 or a longer region between IBS1–2 and FBS2–3 is inhibitory for *DARS2* activation, even if the direction of DNA bending is preserved. Thus, it is conceivable that the location of IBS1–2 and the DNA binding direction would be important for functional assembly of complexes containing IHF, Fis and DnaA bound to *DARS2*.

In addition, FBS2–3 was translocated by 5 or 10-bp toward IBS1–2, resulting in traFBS-L5 and –L10 (Supplementary Figure S6A). Whereas the *DARS2* activity of *DARS2* traFBS-L5 was considerably inhibited, the *DARS2* activity of traFBS-L10 was only moderately affected (Supplementary Figure S6B).

These results support the idea that DNA bending at specific sites is important for *DARS2* activation. However, we cannot exclude the possibility that DnaA bound to the core DnaA boxes interacts with IHF and Fis bound to IBS1–2 and FBS2–3, and for this interaction to occur, the precise locations of the binding sites would be important.

### DnaA oligomer formation is crucial for *DARS2*-mediated ADP dissociation

**Figure 4.**
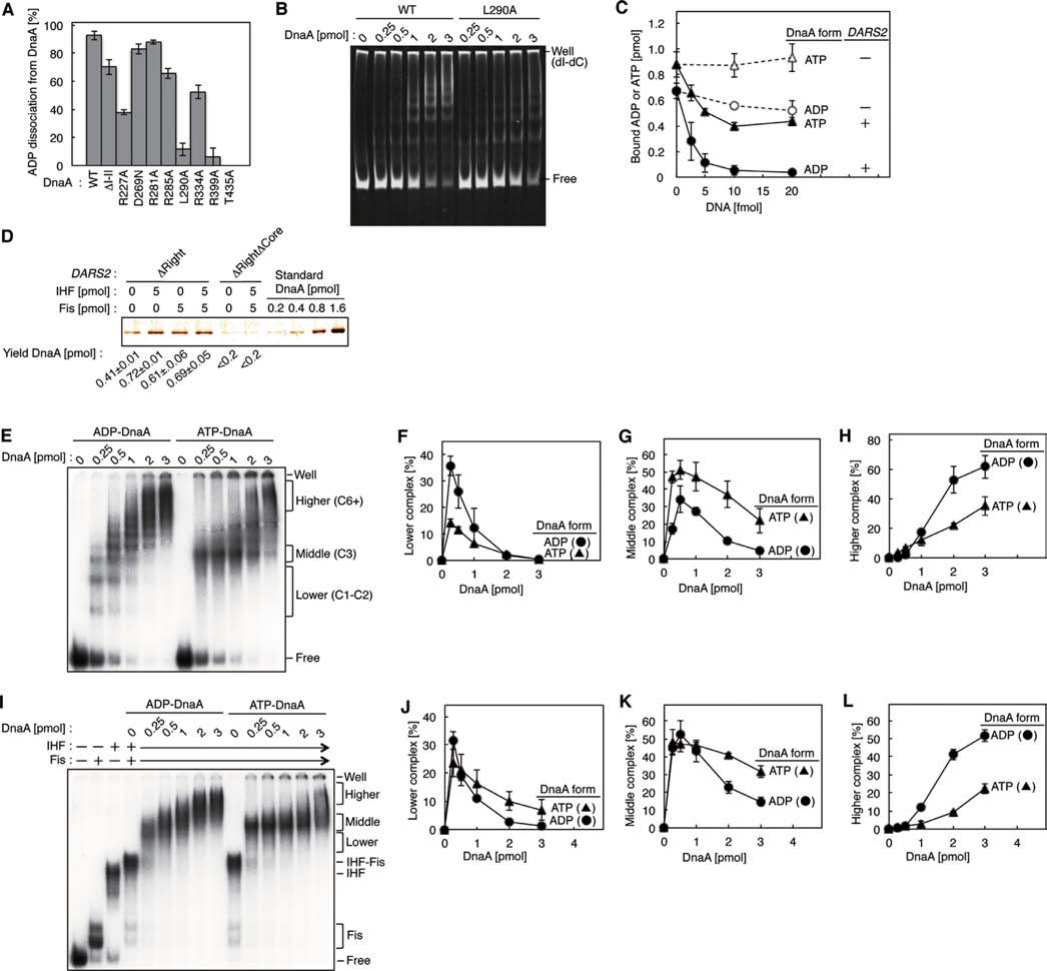
Mechanistic analyses of DnaA oligomer formation on *DARS2*. (**A**) Analysis of DnaA mutants. Wild-type and mutant forms of [^3^H]ADP–DnaA were incubated as described for Figure 2C, with or without 5 fmol of pOA61. Average values and errors of ADP dissociation from DnaA [%] are shown. (**B**) Electrophoretic mobility shift assay using DnaA AID-2 mutant. Indicated amounts of the ADP forms of wild-type DnaA or the DnaA AID-2 mutant L290A were incubated at 30°C for 5 min in dissociation buffer containing *DARS2*ΔRight DNA (15 fmol) and poly (dI-dC) (25 ng), followed by 5% PAGE and GelStar staining. Well; gel well, Bound; protein-bound DNA, Free; protein-free DNA. Poly (dI-dC) remained in the gel well. (**C**) Dissociation of ATP or ADP from DnaA. [α-^32^P]ATP–DnaA (▴,Δ) or [^3^H]ADP–DnaA (•,○) (2 pmol) was incubated with pOA61 (•,▴) or pACYC177 (○, Δ) as described above. Two independent experiments were done for each assay, and both data and mean values are shown. (**D**) Pull-down assay. ADP–DnaA (5 pmol) and 5’-biotinylated DNA (0.5 pmol) of *DARS2*ΔRight or *DARS2*ΔRightΔCore were co-incubated with or without IHF and Fis. DNA-bound DnaA was collected using magnetic beads and was quantitatively analyzed by SDS-PAGE and silver staining. *DARS2*ΔRightΔCore was a *DARS2*ΔRight derivative bearing ΔCore (Figure 1C). Two independent experiments were done, and both data and the mean values are shown for the recovered DnaA. (**E**–**H**) Electrophoretic mobility shift assay using *DARS2*ΔRight/subFBS1. ADP/ATP–DnaA was incubated at 30°C for 5 min in dissociation buffer containing [^32^P]*DARS2*ΔRight/subFBS1 DNA (15 fmol) and poly (dI-dC) (25 ng), followed by 5% PAGE and phosphorimaging (E). Intensities of the signals corresponding to lower (C1–C2) (F), middle (C3) (G) and higher-order multimers (C6+) (H) on the gel image were quantified, and relative levels [% of total] were plotted. Two independent experiments were done, and both data and mean values are shown. (**I**–**L**) Electrophoretic mobility shift assay using *DARS2*ΔRight/subFBS1. Similar experiments were performed in the presence of IHF (0.6 pmol) and Fis (0.3 pmol) (I), followed by analysis of the signals (J–L). Two independent experiments were done, and both data and mean values are shown.

To elucidate the formation of the DnaA complex on *DARS2*, we analyzed DnaA mutants using the *in vitro* reconstituted system described above. DnaA consists of four functional domains: domain I contains specific binding sites for DnaB and self-dimerization; domain II is a flexible linker; domain III is the AAA+ domain; and domain IV is the DnaA box–binding region (1–3). As in the case of *DARS1* (18), DnaA domains I–II were dispensable for the *DARS2*–mediated ADP dissociation, whereas the R399 and T435 residues in domain IV, which are required for DNA binding (29,30), were crucial for dissociation (Figure 4A).

DnaA domain III contains several residues characteristic of the AAA+ family of ATPases: Box VII R281, Arg-finger R285, AID-1 R227 and AID-2 L290. These residues, which are crucial for formation of active initiation complexes, reside on the surface of the domain and are crucial for specific inter-DnaA protomer interactions in initiation complexes (1,7,22). In *DARS2*–mediated ADP dissociation from DnaA, DnaA R281A was active, and DnaA R285A had moderately reduced activity (Figure 4A), similar to the case for *DARS1* (18). By contrast, the R227A and L290A mutants were severely impaired (Figure 4A), suggesting that inter-DnaA interactions via these residues are crucial for *DARS2*–mediated ADP dissociation. Consistently, DnaA oligomerization on *DARS2* was severely inhibited by the L290A mutation (Figure 4B).

In DnaA, the AAA+ sensor I residue D269 and sensor II residue R334 are important for stable nucleotide binding and ATP hydrolysis, respectively (13,31). DnaA D269N was active for *DARS2*–mediated ADP dissociation (Figure 4A and Supplementary Figure S7A). We found that the addition of poly (dI-dC) to these reactions stimulated ADP dissociation from wild-type DnaA by *DARS2*, but not significantly by *DARS1* (Supplementary Figure S7A and B). Even in the absence of poly (dI-dC), DnaA D269N was basically active in ADP dissociation by *DARS2* (Supplementary Figure S7A). ADP dissociation of DnaA D269N by *DARS1* was inefficient in the absence of poly (dI-dC), consistent with our previous data (18). In contrast, DnaA R334A was moderately impaired in *DARS2*-mediated ADP dissociation even in the presence of poly (dI-dC) (Figure 4A), as in the case of *DARS1* (18). These results suggest the existence of both common and distinct modes of inter-DnaA interactions on *DARS1* and *DARS2*.

### Dissociation of ATP is less efficient than that of ADP

To confirm the specificity of ADP dissociation, we compared the dissociation of ADP and ATP from DnaA by *DARS2*. In the reconstituted *DARS2* reaction, ATP dissociation from DnaA was less efficient than ADP dissociation (Figure 4C), suggesting that ATP–DnaA forms oligomers that impede nucleotide dissociation.

### Efficient formation of higher-order complexes of ADP–DnaA on *DARS2*

To assess the roles of IHF and Fis in DnaA assembly on *DARS2*, we first performed pull-down assays using ADP–DnaA and biotinylated *DARS2*ΔRight DNA, the minimal *DARS2* sequence (Figure 1C). Yields of biotinylated DNA and DnaA were 0.15–0.20 pmol (i.e. 30–40% of input DNA) and 0.40–0.42 pmol, respectively (Figure 4D). *DARS2*ΔCore was inactive in DnaA binding (Figure 4D). These results indicate that the core region binds two or three molecules of ADP–DnaA, as predicted from the *DARS2* core sequence. Notably, inclusion of IHF, Fis or both proteins increased yields of DnaA to 0.6–0.7 pmol when *DARS2* core was present, suggesting that IHF and Fis allow binding of at least three to five ADP–DnaA molecules to a single molecule of *DARS2*.

Next, we performed the electrophoretic mobility shift assay to investigate further the formation of DnaA oligomers on *DARS2*. To exclude the effect of FBS1, which is dispensable for ADP dissociation from DnaA (Supplementary Figure S5), we used *DARS2*ΔRight bearing the subFBS1 mutation. In the absence of IHF and Fis, low levels of ADP–DnaA formed smaller complexes than identical levels of ATP–DnaA (C1–C2; Figure 4E–H). This may be related to the propensity that ATP–DnaA forms homomultimers on *oriC* more efficiently than ADP–DnaA (2,3,22). However, high levels of ADP–DnaA formed larger complexes more efficiently than identical levels of ATP–DnaA (C3 and greater; Figure 4E–H). Over a wide range of protein concentrations, ATP–DnaA formed complexes of intermediate size that were more abundant than those formed by ADP–DnaA (C3–C4; Figure 4E–H). Notably, even in the presence of IHF and Fis, ADP–DnaA formed larger complexes more efficiently than ATP–DnaA (Figure 4I–L). Also, the fraction of complexes remaining in the wells was considerably reduced by IHF and Fis. These results are consistent with the results of the pull-down assays (Figure 4D) and the idea that IHF and Fis stimulate ordered ADP–DnaA assembly on *DARS2*.

### *DARS2*-mediated stimulation of initiation *in vivo* requires IHF and Fis

**Figure 5.**
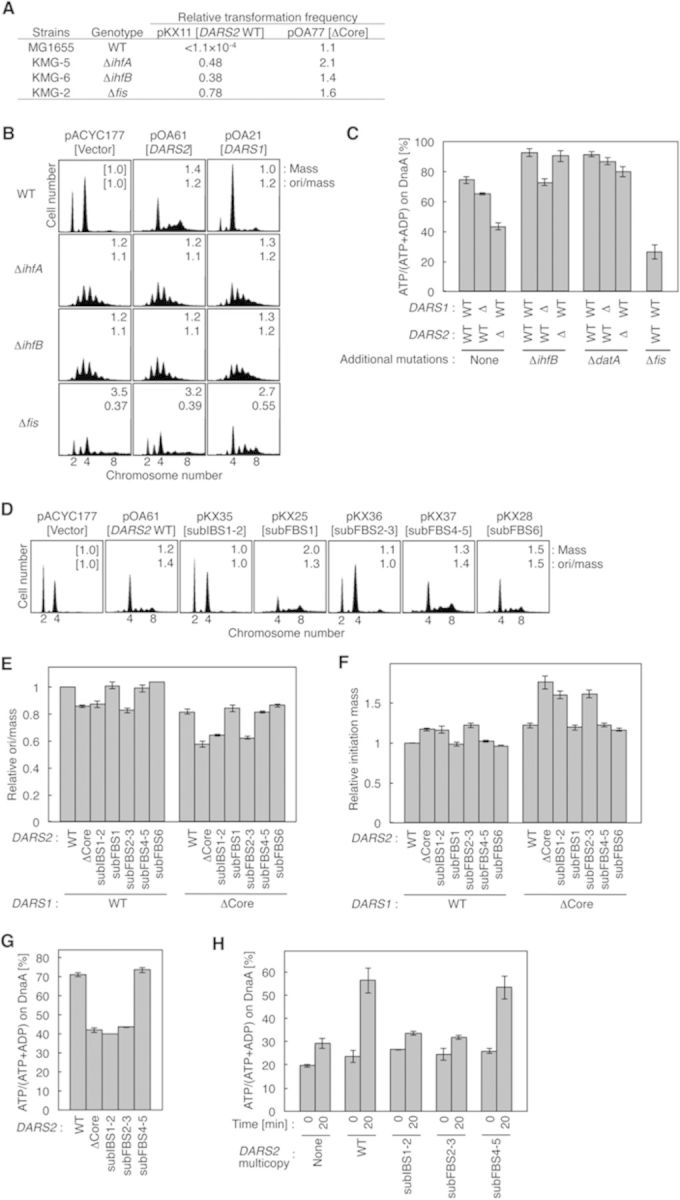
*In vivo* roles for IHF and Fis in *DARS2* activation. (**A**) Transformation inhibition. Strains MG1655 (WT), KMG-5 (Δ*ihfA*), KMG-6 (Δ*ihfB*) and KMG-2 (Δ*fis*) were transformed with pBR322 (vector), pKX11 (*DARS2* WT) or pOA77 (*DARS2*ΔCore) and incubated at 37°C for 12 h on LB-ampicillin agar plates. Transformation efficiencies of pKX11 and pOA77, relative to those of pBR322, are shown. (**B**) Flow cytometry analysis using Δ*ihf* and Δ*fis* cells. MG1655, KMG-2, KMG-5 and KMG-6 cells bearing pACYC177 (vector), pOA61 (*DARS2*) or pOA21 (*DARS1*) were grown at 37°C in supplemented M9-ampicillin medium, and then subjected to flow cytometry analysis as described for Figure 1C. The numbers inserted in the histograms indicate mean cell mass and ori/mass relative to that of MG1655 cells bearing pACYC177. (**C**) Cellular ATP–DnaA levels. The following strains were grown at 37°C; MK86 (WT), MIT47 (*DARS1*ΔCore), MIT86 (*DARS2*ΔCore), KX97 (Δ*ihfB*), KX101 (Δ*ihfB DARS1*ΔCore), KX90 (Δ*ihfB DARS2*ΔCore), KX83 (Δ*datA*), KX179 (Δ*datA DARS1*ΔCore), KX102 (Δ*datA DARS2*ΔCore) and KX29 (Δ*fis*). Error bars represent SD from three independent experiments except for KX29. Two independent experiments were done for KX29, and both data and mean values are shown. (**D**) Flow cytometry analysis using pOA61 derivatives. MG1655 cells bearing pACYC177 (vector), pOA61 (*DARS2* WT) or the indicated pOA61-derivative *DARS2* mutant plasmid were analyzed by flow cytometry as described for Figure 1C. Ratios of mean cell mass and ori/mass are shown in the histograms. (**E**) The ori/mass ratios of the chromosomal *DARS2* mutant cells. Cells of the MG1655 (*DARS1*, WT; *DARS2*, WT) strain and its derivatives bearing the indicated *DARS2* mutations in the presence or absence of the *DARS1*ΔCore mutation were analyzed by flow cytometry. Ratios of two or four *oriC* in each strain were calculated. The ori/mass ratios are shown as relative values to those of MG1655, which was used as a standard. (**F**) The initiation mass of *DARS2* mutant cells. The initiation masses relative to that of MG1655 (WT) were deduced from the flow cytometry data obtained above. (**G**) Cellular ATP–DnaA levels of IBS and FBS mutants. Cells of the KX41 (*rnhA*::Tn*3* Δ*oriC* Δ*hda*) (WT) strain and its derivatives bearing the indicated *DARS2* mutations were analyzed as described for Figure 5C. Two independent experiments were done, and both data and mean values are shown. (**H**) *DARS2*-medicated ATP-DnaA regeneration activity *in vivo*. KA474 (*dnaN59*) cells bearing pACYC177 (None), pOA61 (WT) or the indicated pOA61-derivative *DARS2* mutant plasmid were incubated in TG-ampicillin medium containing [^32^P]orthophosphate at 28°C until the A_660_ reached 0.2, transferred to 42°C in the presence of 150 μg/ml chloramphenicol (Time 0) and further incubated for 20 min, followed by immunoprecipitation and thin-layer chromatography. Two independent experiments were done for each assay, and both data and mean values are shown.

To investigate the *in vivo* roles of IHF and Fis in *DARS2* activation, we first assessed the sensitivity of cells harboring mutations in *ihfA*, *ihfB* or *fis* (encoding IHF α, IHF β and Fis, respectively) to *DARS2* oversupply. Introduction of pKX11 (pBR322 bearing full-length *DARS2*) severely inhibited colony formation of wild-type cells in an *oriC-* and *dnaA-*dependent manner (18). In contrast to wild-type cells, mutant cells bearing pKX11 formed stable colonies at a level similar to those bearing pOA77 [pBR322-*DARS2*ΔCore] (Figure 5A).

Next, we used flow cytometry to analyze the initiation in mutant cells bearing pACYC177-*DARS2* (pOA61) or pACYC177-*DARS1* (pOA21). Whereas MG1655 cells bearing the parental vector pACYC177 contained two or four origins, those bearing pOA61 predominantly contained four or eight origins, and some cells contained five or seven origins as a result of asynchronous extra initiations (Figure 5B). These cells were larger on average than cells bearing the parental vector, possibly because overinitiation impeded cell division (24). Introduction of pOA21 increased the level of four-origin cells relative to the level in cells bearing the parental vector, indicating that pOA21 moderately stimulated initiation (Figure 5B). These data are consistent with our previous findings (18).Δ*ihfA* or Δ*ihfB* cells bearing pACYC177 initiated replication asynchronously and predominantly contained three to five origins (Figure 5B), in agreement with a previous report (32) and with the idea that IHF plays positive and negative roles in initiation at *oriC* and *datA*. Introduction of pOA61 did not change the distribution of origin number in either *ihf* mutant (Figure 5B). By contrast, mutant cells bearing pOA21 predominantly contained four to six origins (Figure 5B); even the number of cells containing more than six origins was higher in mutant cells bearing pOA21 than in those bearing pACYC177. The reduction in the number of cells containing three origins could have been a consequence of the increase in the number of cells containing four and more origins. These results are consistent with the idea that *DARS2* activity, but not *DARS1* activity, depends on IHF.Δ*fis* cells bearing pACYC177 also initiated replication asynchronously, and the ratio of origin number to mean cell mass (ori/mass) was reduced to 0.37 relative to the ratio in MG1655, as reported in previous studies (20,21). The ori/mass ratio is used to indicate the relative level of initiation activity (18,33). pOA61 did not affect initiation in Δ*fis* cells, whereas pOA21 stimulated initiation (Figure 5B). These results support the idea that Fis, along with IHF, is specifically required for activation of *DARS2* but not for the activation of *DARS1*. The doubling times of the cells used in these experiments were 32 ± 4 min, which was essentially the same for all strains.

### *DARS2* requires IHF and Fis for ATP–DnaA regeneration *in vivo*

Next, we analyzed ATP–DnaA regeneration activity in Δ*ihfB*, Δ*datA* and Δ*fis* cells. To eliminate effects of RIDA (Figure 1A), we used strain MK86 [Δ*oriC* Δ*rnhA* Δ*hda*] as a common background in these experiments. Δ*hda* inhibits RIDA, resulting in an elevated level of ATP–DnaA and overinitiation at *oriC*, ultimately inhibiting cell growth (12,34). Introduction of Δ*oriC* to these cells represses the lethality of the Δ*hda* mutation in the presence of Δ*rnhA*, a mutation that activates alternative DnaA-independent origins (35). DnaA was isolated by immunoprecipitation (IP) from lysates of ^32^P-labeled cells, and DnaA-bound nucleotides were eluted and quantitated by thin-layer chromatography, as described previously (11,12,18). In these experiments, nucleotides were labeled and DnaA-bound, labeled nucleotides were analyzed (Supplementary Figure S8). In MK86 cells, the ATP–DnaA level (i.e. the percentage of DnaA in the ATP-bound form) was high (72%), but this level decreased upon deletion of *DARS1* or *DARS2* (Figure 5C), as we previously demonstrated (11,12,18).

The ATP–DnaA levels in Δ*ihfB* or Δ*datA* cells were 90–95%, higher than in the parental MK86 cells (Figure 5C), which is consistent with our previous report showing that deletion of *ihfB* or *datA* elevates the ATP–DnaA level because of DDAH inactivation (17). Notably, the ATP–DnaA level in Δ*ihfB* cells was not affected by Δ*DARS2*, but it was decreased to 73% by Δ*DARS1* (Figure 5C). These results support the idea that *DARS2*, but not *DARS1*, requires IHF for ATP–DnaA regeneration *in vivo*. Furthermore, the ATP–DnaA level in Δ*datA* cells was decreased to 80% by deletion of *DARS2* and was decreased slightly by Δ*DARS1* (Figure 5C), suggesting that IHF activates *DARS2* in a *datA*-independent manner.

In addition, deletion of *fis* decreased the ATP–DnaA level to 26% (Figure 5C), supporting the idea that Fis, as well as IHF, is crucial for ATP–DnaA regeneration *in vivo* (see also Discussion). Growth rates and IP yields were indistinguishable among Δ*ihfB*, Δ*datA* and Δ*fis* cells.

### IBS1–2 and FBS2–3 are required for ATP–DnaA regeneration *in vivo*

To investigate the roles of IBS and FBS in *DARS2* activation *in vivo*, we first performed flow cytometry analysis of MG1655 cells bearing pOA61 derivatives. As shown in Figure 5D, initiation was stimulated by pOA61, pKX25 [subFBS1], pKX37 [subFBS4–5] and pKX28 [subFBS6]. By contrast, initiation was not substantially stimulated or only slightly stimulated by pKX35 [subIBS1–2] or pKX36 [subFBS2–3], respectively, compared to initiation stimulated by pOA61. The slight stimulation seen with pKX36 might have been a consequence of the basal, non-specific binding activity of Fis (5,19,28).

Next, we used flow cytometry to analyze the ori/mass ratios in chromosomal *DARS2* mutants (Figure 5E). *DARS2*ΔCore cells exhibited significantly reduced ori/mass ratios. *DARS2* subIBS1–2 and subFBS2–3 cells had a reduced ori/mass ratio similar to that of the *DARS2*ΔCore, whereas the ratio was unaffected by subFBS1, subFBS4–5 or subFBS6. Similar results were obtained using cells with the *DARS1*ΔCore background (Figure 5E). Furthermore, we used these data to deduce the initiation mass, the cell size per origin at the time of replication initiation (Figure 5F). Initiation mass is an important cell cycle parameter that indicates the potential for replication initiation (36). For instance, a decrease in initiation activity results in an increase in the initiation mass. The data shown in Figure 5 are consistent with the ori/mass ratios (Figure 5E).

In addition, we analyzed ATP–DnaA levels in IBS and FBS mutants with the MK86 background (Figure 5G). Deletion of the *DARS2* core in this background decreased the ATP–DnaA level from 72 to 42%, as previously demonstrated (18). Unlike *DARS2* subFBS4–5, *DARS2* subIBS1–2 or subFBS2–3 decreased the level to 40–45% (Figure 5G). Taken together, these results indicate that specific binding of IHF and Fis to IBS1–2 and FBS2–3 is required for *DARS2* activation *in vivo*, in agreement with the *in vitro* data.

Based on these results, we investigated ATP–DnaA regeneration *in vivo* in *DARS2* mutants (Figure 5H). We previously demonstrated that ATP–DnaA regeneration is stimulated in a pOA61-dependent manner under conditions where RIDA and *de novo* DnaA synthesis are inhibited (18). RIDA is a major pathway for the hydrolysis of ATP bound to DnaA. *De novo* synthesized DnaA will bind ATP, an abundant molecule in the cell, yielding ATP–DnaA. As described in our previous report (18), it is possible to inhibit RIDA and *de novo* protein synthesis including that of DnaA by introducing a temperature-sensitive mutation (*dnaN59*) into the *dnaN* gene encoding the clamp and adding chloramphenicol to the growth medium. The *dnaN59* mutant growing at 28°C was transferred to 42°C and chloramphenicol was added to the medium. After incubation for 20 min, IP and thin-layer chromatography analysis indicated a higher ATP–DnaA level in cells bearing pOA61 than that in cells bearing pACYC177 (Figure 5H), in agreement with our previous results (18). A slight increase of the ATP–DnaA level in cells bearing pACYC177 after 20-min incubation might be caused by *DARS*s carried on the chromosome. Notably, the results obtained using cells bearing pKX35 [*DARS2*subIBS1–2] or pKX36 [*DARS2*subFBS2–3] were similar to those obtained using cells bearing pACYC177, and the results obtained using cells bearing pKX37 [*DARS2*subFBS4–5] were similar to those obtained using cells bearing pOA61. In addition to the data presented in Figure 5C, these results support the idea that specific binding of IHF and Fis to *DARS2* promotes ATP–DnaA regeneration *in vivo*.

### Cell cycle-coordinated binding of IHF to *DARS2*

To investigate whether IHF binding to *DARS2* is coordinated with the replication cycle, we performed ChIP or chromatin affinity precipitation (ChAP) assays using the *dnaC2* mutant, which is temperature sensitive for helicase loading and replication initiation, but not for the progression of established replisomes at 38°C. When *dnaC2* cells grown at 30°C are shifted to 38°C for 90 min, chromosomes are completely replicated, but no new initiations take place. When the cells are returned to 30°C, synchronized initiations occur within 5–10 min after the temperature shift, followed by a second round of initiation at 25–35 min after the shift (11,17,37).

**Figure 6.**
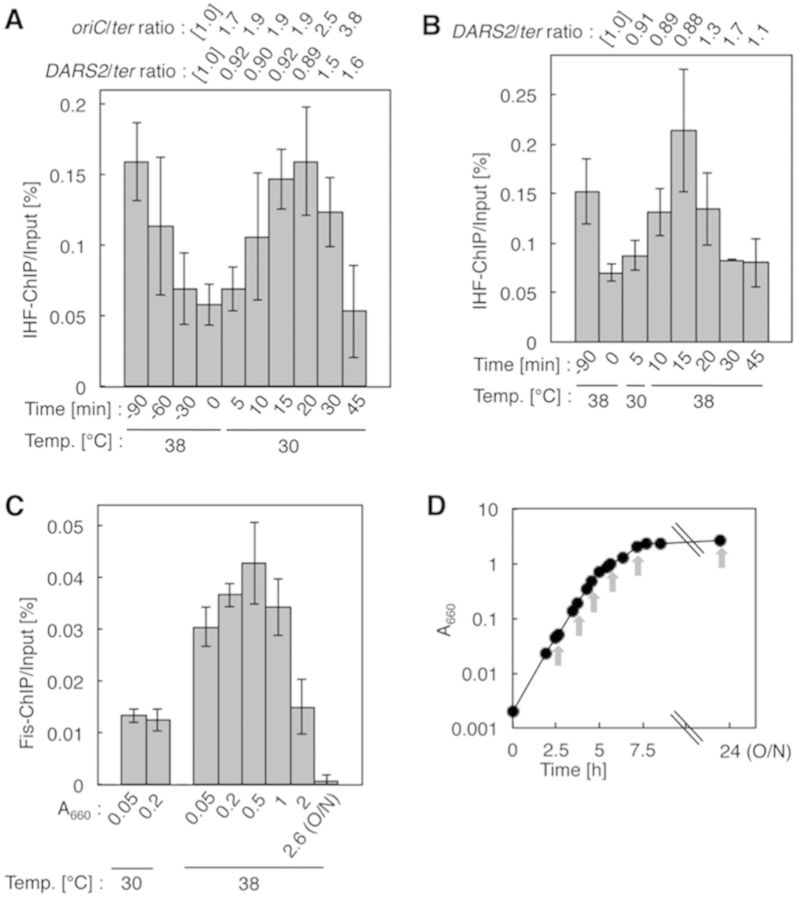
Cell cycle- and growth phase-coordinated regulation of *DARS2*. (**A**, **B**) IHF-ChIP of *DARS2*. KYA018 (*dnaC2*) cells growing at 30°C were transferred to 38°C and incubated for 90 min. The cells were then transferred to 30°C (Time 0) and incubated for 5 min, followed by further incubation at 30°C (A) or 38°C (B). The relative *DARS2* levels before (Input) and after (IHF-ChIP) IP using anti-IHF antiserum were determined using real-time qPCR, yielding the ChIP/Input ratio [expressed as%]. Error bars represent SD from at least three independent experiments. In addition, cellular levels of *oriC, DARS2* and *ter* in the Input samples were quantified, and the relative ratios of *oriC/ter* and *DARS2*/*ter* are expressed relative to the ratio at 0 min (defined as 1). (**C**, **D**) Fis-ChIP of *DARS2*. MG1655 cells were incubated at 30 or 38°C in supplemented M9 medium. At the indicated A_660_, samples were withdrawn for determination of the Fis-ChIP/Input ratio [expressed as%] (C). Error bars represent SD from at least three independent experiments. O/N, overnight incubation. Growth curve at 38°C (D). Arrows: sampling times.

In synchronized cell cultures, *oriC*/*ter* (replication terminal locus) ratios suggested that the first and second initiations occurred respectively within 5 and 30–45 min after the shift to 30°C (Figure 6A). Fluctuations in IHF binding to *oriC* were also consistently observed (Supplementary Figure S9A), as reported previously (17,38). IHF bound to *DARS2* was dissociated before the first initiation, bound again to *DARS2* between the first and second rounds of initiation and then dissociated again (Figure 6A). ChAP experiments using His-tagged IHF yielded similar results (Supplementary Figure S9B and C). These changes are consistent with the changes in ATP–DnaA levels we observed previously (11): the ATP–DnaA level reached its maximum level before the first round of initiation, decreased to the basal level after initiation and then increased again before the second round of initiation. It is logical to assume that IHF dissociates from *DARS2* before the first round of initiation, because the ATP–DnaA level is maximal at this time. IHF binding to *DARS2* after the first initiation occurs at an appropriate time to increase the ATP–DnaA level for the second initiation. *DARS2*/*ter* ratios, which indicate the time of duplication of *DARS2* during replication, suggested that duplication of *DARS2* is not required for IHF binding during this period (Figure 6A). The basal level of IHF–*DARS2* binding might be a consequence of the non-specific, unstable binding of IHF in the *DARS2* region.

Similar results were obtained in synchronized cultures in which the second round of initiation was inhibited by a temperature up-shift (Figure 6B) or addition of rifampicin (Supplementary Figure S9D). These results indicate that IHF binding to and dissociation from *DARS2* is independent of replication initiation as well as transcription, and are consistent with the idea that the IHF–*DARS2* interaction is coordinated with the cell cycle. These features of IHF interaction at *DARS2* are similar to those observed at *datA* (17) (see Discussion).

### Growth phase-dependent binding of Fis to *DARS2*

We performed similar cell-cycle analyses of Fis binding. Unlike IHF-*DARS2* binding, Fis-*DARS2* binding was essentially sustained throughout the replication cycle (Supplementary Figure S9E), although a slight loss in binding might have occurred temporarily after initiation (i.e. at 45 min of Supplementary Figure S9E). When the cultures were incubated at 30°C for a limited time, Fis–*DARS2* binding temporarily decreased. These changes cannot be interpreted as being linked to the replication cycle, however, because Fis–*DARS2* binding was considerably lower in cells incubated at 30°C (Figure 6C). ChIP experiments, which used 3 M urea-denaturing conditions after the crosslinking step, yielded consistent results (Supplementary Figure S9F).

Expression of Fis depends on growth rate and growth phase: it is abundant (10 000–50 000 molecules/cell) in early exponential phase, but the levels decrease to <100 molecules/cell from late exponential phase to stationary phase (5,39). Fis–*DARS2* binding showed similar kinetics: binding was high in exponential phase and significantly lower in late exponential phase to stationary phase (Figure 6C and D). These results support the idea that Fis is the determinant of growth phase-coordinated activation of *DARS2*. By contrast, IHF is abundant throughout all growth phases (5), and we observed that its binding to *DARS2* was basically independent of growth phase (Supplementary Figure S9G).

### Evolutionary conservation of IBS1–2 and FBS2–3

Whereas IHF is broadly conserved across all eubacterial species, Fis is conserved specifically among γ-proteobacterial species related to *E. coli* (40), as is the *DARS2* core sequence (18). The IBS1–2 and FBS2–3 sequences are highly conserved in representative bacterial species in which Fis and IHF homologs are conserved and in which the *DARS2* core has a genomic position similar to that in *E. coli* (Supplementary Figure S10A). These observations suggest that the regulatory mechanism for *DARS2* is shared among these species, including some pathogens.

## DISCUSSION

**Figure 7.**
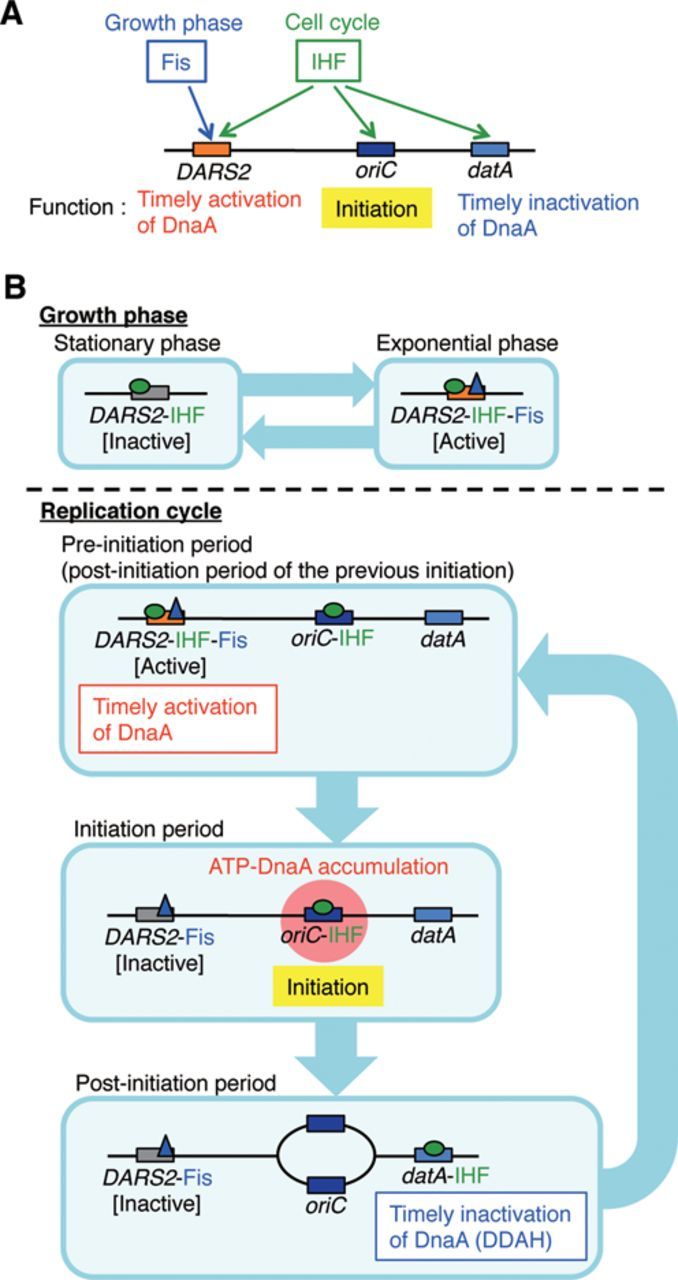
Regulated activation of *DARS2* by IHF and Fis in the DnaA cycle. (**A**) A schematic view of the roles for IHF and Fis in regulatory systems for *DARS2, oriC and datA*. See text for details. (**B**) A model of the timely regulation of *DARS2* by IHF and Fis. In exponential phase cells, Fis binds to *DARS2*. In the pre-initiation stage, the time when the ADP–DnaA level is high, IHF binds to *oriC* and *DARS2* in a cell cycle-coordinated manner, and the *DARS2*–IHF–Fis complex increases the level of ATP–DnaA. Next, the elevated ATP–DnaA level allows the IHF-bound *oriC* to initiate replication, at which point IHF dissociates from *DARS2*. In the post-initiation stage, IHF binds to *datA*, activating DDAH. IHF then dissociates from *datA* in a cell cycle-coordinated manner, and re-binds to *DARS2* and *oriC* for the next round of initiation. For simplicity, only single molecules of IHF and Fis are shown to bind to *DARS2* in this figure. In growing cells, the pre-initiation stage overlaps the post-initiation stage of the previous round of initiation.

The primary regulatory system responsible for the temporal increase in the ATP–DnaA level during the cell cycle has remained elusive. However, in this study, we revealed that simultaneous binding of IHF and Fis to *DARS2* activates ATP–DnaA production (Figure 7A). In contrast to initiation at *oriC*, HU cannot substitute for IHF in *DARS2* activation, indicating a crucial role for IHF in this regulation. Cell-cycle and growth-phase analyses revealed that IHF binds to specific sites within *DARS2* in a cell cycle-coordinated manner that is independent of DNA replication, whereas Fis binds specifically in the exponential phase. In addition, *DARS2*–mediated ADP dissociation from DnaA was more efficient than ATP dissociation, which is consistent with the more efficient formation of higher-order complexes by ADP–DnaA and *DARS2*. Taken together, these results demonstrate that activation of *DARS2* is regulated in coordination with the cell cycle and growth phase via temporal changes in the binding of IHF and Fis.

IHF plays multiple crucial roles in the regulation of initiation. First, IHF binds to *oriC* at the pre-initiation stage (Figure 7A and B), stimulating DnaA assembly on *oriC* and unwinding of the duplex unwinding element (8,9,25,38). The requirement for IHF in replication was first demonstrated using the plasmids pSC101 and R6K, whose origins bear IBS (41,42). Second, IHF binds to *datA*, and this interaction is required for activation of DDAH (Figure 7A and B). In replication cycle-synchronized cultures, IHF–*datA* binding occurs only for a short time at a post-initiation stage: levels of binding increase immediately after initiation and peak 15 min later, followed by rapid dissociation (17). Third, as demonstrated in this study, IHF binds to *DARS2*, activating ATP–DnaA regeneration (Figure 7A and B). At 30°C, IHF–*DARS2* binding began to increase 5 min after initiation, peaked within 20 min and was sustained for an additional 10 min, followed by dissociation; binding levels returned to the basal level at 45 min. This fluctuation is consistent with the observation that under the same conditions, the second initiation occurs 30–45 min after the first, as well as with the fact that the cellular ATP–DnaA level peaks around the time of replication initiation. Thus, IHF binding to *DARS2*, *oriC* and *datA* is regulated differently, resulting in cell cycle-coordinated regulation of initiation (Figure 7A and B). In other words, IHF plays both positive and negative roles in initiation that are regulated temporally and by the locations of DNA binding during the cell cycle.

The dynamic interactions of IHF–*DARS2* are independent of replication initiation: these events occur in a timely manner even under conditions in which the second round of initiation is inhibited (Figure 6B and Supplementary Figure S9D). This observation supports the idea that a mechanism regulating dynamic IHF–*DARS2* interactions is coupled with a cell cycle-regulatory pathway that is epistatic to replication regulation. Similarly, temporal regulation of the IHF–*datA* interaction also would be regulated in a cell cycle-coupled manner (17). A recent study, which applied genome conformation capture analysis to the *E. coli* genome, suggested that a *DARS2* region spatially interacts with *oriC* only in growing cells (43), consistent with the existence of a possible mechanistic link in the timely delivery of IHF and ATP–DnaA between *oriC* and *DARS2*. In addition, a specific factor that dissociates IHF–*DARS2* complexes, if present, might be crucial for cell cycle-coordinated replication regulation. Recently, we found a crude protein extract that contains IHF–*DARS2* dissociating activity, but not Fis–*DARS2* dissociating activity.

The molecular mechanism responsible for initiation stimulation by Fis has remained obscure (20,21). A previous report suggests that Fis binding to *oriC*, even if it occurs, does not affect replication initiation *in vivo* (44). The present study provides a reasonable solution to the long standing question of how Fis contributes to initiation; the present solution is that Fis actives *DARS2* in the presence of IHF, stimulating ATP–DnaA production and initiation. Fis binds to *DARS2* during exponential phase, but not late stationary phase (Figure 6C), indicating that Fis determines the growth-phase specificity of *DARS2* activity. This might be related to the dramatic fluctuations in the cellular level of Fis, which peaks early in exponential phase and disappears in late stationary phase. Also, depending on the growth rate, the cellular level of Fis might regulate *DARS2* activity. Deletion of *fis* causes a severe reduction in the cellular ATP–DnaA level (Figure 5C). In addition to providing a mechanism for Fis-dependent stimulation of initiation, these data suggest the existence of a Fis-dependent third *DARS*, which is yet to be unidentified.

Specific residues for inter-DnaA interactions, as well as those for DNA binding, are crucial for *DARS2*–mediated ADP dissociation. The basic AID-1 (R227) and hydrophobic AID-2 (L290) residues are important for ADP dissociation (Figure 4A). As in the case of initiation complexes (22), these residues might stabilize inter-DnaA interactions, leading to construction of functional higher-order complexes. In support to this idea, AID-2 stimulated DnaA oligomerization on *DARS2* (Figure 4B). The requirements for other DnaA AAA+ residues for *DARS2* are similar to those for *DARS1*, suggesting that the mechanistic principles are conserved between the two loci.

In addition, the requirement for a DNA region that binds to IHF and Fis during *DARS2* activation is consistent with the fact that in *DARS1*, a specific DNA region flanking the core DnaA boxes stimulates ADP dissociation from DnaA (18). In the case for *DARS2*, the DNA conformational change induced by binding of IHF and Fis might be crucial for the interaction of this region with the DnaA complex formed at core DnaA boxes (Supplementary Figure S10B). Thus, it is plausible that, as in the case of *DARS1* complexes (18), IHF-Fis-bound *DARS2* causes changes in inter-DnaA interactions in the DnaA complex that lead to the conformational changes in ADP–DnaA protomers that induce ADP dissociation. The resultant apo-DnaA would bind ATP after its release from *DARS2*, yielding initiation-competent ATP–DnaA (Figure 2). ATP–DnaA is less active for higher complex formation on *DARS2* than ADP–DnaA (Figure 4), which might allow preferential binding of ADP–DnaA to *DARS2*, enhancing cyclic reactivation of DnaA (Supplementary Figure S10B).

In eukaryotes, ORC (origin recognition complex) is regulated by its nucleotide-bound state, and its major subunits exhibit structural similarity to the DnaA AAA+ domain (45). Eukaryotic HMG (high mobility group) proteins are thought to play roles similar to those of the bacterial nucleoid-associated proteins, i.e. to promote DNA conformational changes (5). Yeast NHP6-A, a member of the HMG family, complements defects in nucleoid compaction and in λ-excision caused by the lack of nucleoid-associated proteins in *E. coli* (46). Although sequences homologous to *DARS* have not been reported in eukaryotic cells, this does not exclude the possibly that nucleotide-bound states of ORC or eukaryotic AAA+ proteins are regulated by HMG proteins and *DARS2*-like sequences in these cells.

## SUPPLEMENTARY DATA

Supplementary Data are available at NAR Online.

SUPPLEMENTARY DATA
