# Supplementary material for: Timely binding of IHF and Fis to DARS2 regulates ATP–DnaA production and replication initiation
Source: Nucleic Acids Res. 2014 Nov 6;42(21):13134–49. doi: 10.1093/nar/gku1051 (PMC4245941; doi:10.1093/nar/gku1051)
Supplement: SUPPLEMENTARY DATA [file supp_42_21_13134__index.html]

Timely binding of IHF and Fis to DARS2 regulates ATP–DnaA production and replication initiation — Timely binding of IHF and Fis to DARS2 regulates ATP–DnaA production and replication initiation — SUPPLEMENTARY DATA 

# Timely binding of IHF and Fis to *DARS2* regulates ATP–DnaA production and replication initiation

## SUPPLEMENTARY DATA

**Files in this Data Supplement:**

- SUPPLEMENTARY DATA
