# Supplementary material for: Timely binding of IHF and Fis to DARS2 regulates ATP–DnaA production and replication initiation
Source: Nucleic Acids Res. 2014 Nov 6;42(21):13134–49. doi: 10.1093/nar/gku1051 (PMC4245941; doi:10.1093/nar/gku1051)

**Supplementary Data**

**Timely binding of IHF and Fis to *DARS2* regulates ATP–DnaA production and replication initiation**

Kazutoshi Kasho, Kazuyuki Fujimitsu, Toshihiro Matoba, Taku Oshima and Tsutomu Katayama

## Supplementary Materials and Methods

***Proteins, DNA, and* E. coli *strains***

Wild-type or mutated DnaA proteins were overexpressed and purified as we previously described (1, 2). A crude protein extract (Fr II) was prepared using lysates of exponentially growing cells and ammonium sulfate precipitation, as described (3). IHF was overexpressed with arabinose in MC1061 cells and purified, as described (4–6). For construction of C-terminally 6His-tagged Fis-overproducer, the *fis* gene was amplified by PCR using primers Fis-1 and Fis-3, digested by NdeI and XhoI, and ligated at the NdeI and XhoI sites of pET21a, resulting in pET-Fis6H. The tagged Fis protein was overexpressed in BL21(λDE3) cells, purified using $Ni^{2+}$-NTA agarose column and stored in elution buffer containing 40 mM Hepes-KOH (pH7.6), 10 mM β-mercaptoethanol, 0.1 mM EDTA, 500 mM NaCl, 10% (v/v) glycerol, 250 mM imidazole. Purity of each protein was >90% as judged by SDS-PAGE and Coomassie Brilliant Blue staining.

Plasmid pOA61 is a pACYC177 derivative bearing *DARS2* (3). *DARS2*ΔRight and Δ(Right+21) DNA fragments were amplified by PCR using pOA61 as a template and a set of primers MutH-4 and MutH-11, and MutH-4 and MutH-17, respectively, digested by HindIII and BamHI, and ligated at the HindIII and BamHI sites of pACYC177, resulting in pOA67 and pOA73, respectively. pOA61 derivatives including *DARS2* sequence substitutions were constructed by inside-out PCR using the following primers (Supplementary Table S1): MutH-14 and MutH-15 for pOA71 (ΔCore), MutH-24 and MutH-25 for pOA86 (Δ21), Ksh-16 and Ksh-19 for pKX35 (subIBS1–2), Ksh-3 and Ksh-4 for pKX25 (subFBS1), Ksh-20 and Ksh-21 for pKX36 (subFBS2–3), Ksh-22 and Ksh-23 for pKX37 (subFBS4–5), Ksh-9 and Ksh-10 for pKX28 (subFBS6), Ksh-30 and Ksh-31 for pKX44 (subDnaAboxIV), Ksh-32 and Ksh-33 for pKX45 (subDnaAboxV), Ksh-34 and Ksh-35 for pKX46 (subDnaAboxVI), traIL-U and traIL-L1 for pKX98 (traIBS-L5), traIL-U and traIL-L2 for pKX99 (traIBS-L10), traIR-U1 and traIR-L for pKX100 (traIBS-R5), traIR-U2 and traIR-L for pKX101 (traIBS-R10), traF-U and traF-L1 for pKX102 (traFBS-L5), traF-U and traF-L2 for pKX103 (traFBS-L10), and insIBS-10U and insIBS-L for pKX107 (insIBS). pOA61tet-derivatives for λ Red recombination, as described below, were similarly constructed using the following primers (Supplementary Table S1): Ksh-16 and Ksh-19 for pKX35tet (subIBS1–2), Ksh-3 and Ksh-4 for pKX25tet (subFBS1), Ksh-20 and Ksh-21 for pKX36tet (subFBS2–3), Ksh-22 and Ksh-23 for pKX37tet (subFBS4–5), and Ksh-9 and Ksh-10 for pKX28tet (subFBS6). pSTV28-C-12His carried a chemically synthesized sequence encoding 12xHis and a

plasmid pKD4–derived *frt-kan* region at the multicloning site of pSTV28 (Takara Bio). pKD4 is previously described (7).

A *DARS2* wild-type (WT) fragment (455 bp) for the electrophoretic mobility shift assay was amplified by PCR using pOA61 and primers MutH-2 and MutH-4 (Supplementary Table S1). *DARS2*FP-Upper and -Lower fragments (618 bp) for footprint analysis was amplified by PCR using pOA61 and primers pOA61-FP1 and pOA61-FP2 of which 5'-end was labeled with $^{32}$P. Oligonucleotides for the electrophoretic mobility shift assay (FIS-1U and FIS-1L for FBS1, FISsub-5U and FISsub-5L for subFBS1, F23U and F23L for FBS2-3, subF23U and subF23L for subFBS2-3, F45U and F45L for FBS4-5, subF45U and subF45L for subFBS4-5, FIS-4U and FIS-4L for FBS6, and FISsub-4U and FISsub-4L for subFBS6) were annealed at room temperature overnight. Biotinylated *DARS2*ΔRight and *DARS2*ΔRightΔCore DNA for pull down assay were amplified using pOA61 and pOA71, respectively, and primers 5'-biotinylated MutH-4 and MutH-19 (Supplementary Table S1). *DARS2*ΔRight DNA (243 bp) for the electrophoretic mobility shift assay was amplified using pOA61. *DARS2*ΔRight/subFBS1 DNA (243 bp) for the electrophoretic mobility shift assay was amplified using pKX25 (subFBS1) and the primers MutH-4 and MutH-19, and labeled using [γ-$^{32}$P]ATP and T4 polynucleotide kinase. The wild-type *datA* and subDnaAbox2 fragments (991 bp) are previously described (6). The wild-type *DARS1* fragment FK7-7 and FK7-21, a derivative of FK7-7 lacking the core DnaA boxes (ΔCore) are previously described (3).

All *E. coli* strains used in this study are listed in Supplementary Table S2. Δ*ihfA*::*frt-kan*, Δ*ihfB*::*frt-kan,* and Δ*fis*::*frt-kan* derived from the Keio collection were introduced into MG1655 cells using P1 transduction, yielding strains KMG-5, KMG-6, and KMG-2, respectively (Supplementary Table S2). Δ*fis*::*frt-kan* derived from the Keio collection was introduced into MK86 cells using P1 transduction, yielding a strain KX29 (Supplementary Table S2). The chromosomal *ihfB* and *DARS1* core regions were replaced with the *spec* gene using MG1655 cells harboring pKD46 (λ Red expression plasmid) (7) and DNA fragments amplified using pCL1920 (3) as a template and primers Ksh-26 and Ksh-27, and D1spec-U and D1spec-L, respectively, resulting in strains KX95 and KX176, respectively (Supplementary Table S2). The resultant Δ*ihfB*::*spec* region was introduced into MK86 cells using P1 transduction, yielding KX97. Similarly, *DARS1*ΔCore::*spec* was introduced into strains MK86 and KX93, yielding strains KX178 and KX179, respectively. *DARS1*ΔCore::*kan*, which was derived from a strain MIT17 (3), was introduced into KX95 cells, yielding KX101. *DARS2*ΔCore::*spec*, derived from MIT84, was introduced into strains KX31 and KX93 cells, yielding strains KX90 and KX92, respectively. The C-terminally 12xHis-tagged *ihfA* gene (*ihfA-cHis12*) was constructed by introducing a DNA fragments amplified using pSTV28-C-12His and primers TOP1829 and TOP1830 into the C-terminus of the coding region of the chromosomal *ihfA* gene, using BW25113 cells harboring pKD46 (7) (Supplementary Table S2). The amplified pSTV28-C-12His–derived fragment contained the *frt-kan* region and a sequence encoding 12xHis and its flanking Arg-Gly-Ser linker. The resultant *ihfA-cHis12 frt-kan* region was introduced into KYA018 cells using P1 transduction, and the *kan* region was removed by pCP20 (7), yielding SH022.

Chromosomal *DARS2* mutants with IBS- or FBS-substitution were similarly introduced in MG1655 cells harboring pKD46; DNA fragments carrying the *DARS2* mutation and *tet* gene were amplified using pOA61tet for *DARS2* WT-*tet*, pKX35tet for *DARS2*subIBS1-2-*tet*, pKX25tet for *DARS2*subFBS1-*tet*, pKX36tet for *DARS2*subFBS2-3-*tet*, pKX37tet for *DARS2*subFBS4-5-*tet*, and pKX28tet for *DARS2*subFBS6-*tet* and primers D2TET-1 and mutH-2Nosite. The resultant mutations, i.e., *DARS2* WT-*tet,* subIBS1-2-*tet*, subFBS1-*tet*, subFBS2-3-*tet*, subFBS4-5-*tet*, and subFBS6-*tet*, were introduced into MG1655 cells using P1 transduction, yielding strains MIT187, KX53, KX5, KX54, KX55, and KX7, respectively, and were also introduced into MIT17 cells, yielding strains KX58, KX8, KX59, KX60, and KX10, respectively (Supplementary Table S2). For construction of *DARS2-frt-kan* derivatives, the *tet* genes in MIT187, KX53, KX54, and KX55 were replaced using λ Red system with the *frt-kan* gene which was amplified using pTH5 (8) and primers Ksh-14 and Ksh-15. The resultant mutations, i.e., *DARS2* WT-*frt-kan*, subIBS1-2-*frt-kan*, subFBS2-3-*frt-kan*, and subFBS4-5-*frt-kan*, were introduced into a strain MK86, yielding strains KX41, KX68, KX69, and KX70, respectively (Supplementary Table S2).

M9 medium was supplemented with 0.2% glucose, 0.2% casamino acid, and 5 μg/mL thiamine. Also, 50 μg/mL ampicillin was included, if required.

### *Flow cytometry analysis*

Flow cytometry analysis was performed as previously described (3). Typically, cells were grown at 37 °C in supplemented M9 medium until the $A_{600}$ (absorbance at 600 nm) reached 0.1–0.2 (Figures 5B, 5D, and Supplementary Figure S1), followed by further incubation at 37 °C for 4 hr in the presence of 300 μg/mL rifampicin and 10 μg/mL cephalexin for run-out replication. The resultant cells were fixed, stained with SYTOX Green (lifetechnologies) and analyzed with FACS Calibur flowcytometry (BD Biosciences).

### In vitro *reconstitution of DARS2 DnaA-ADP dissociation, ATP-DnaA regeneration, and DnaA cycle with RIDA or DDAH*

In the reconstituted system of *DARS2*-medicated ADP dissociation from DnaA, [$^3$H]ADP-DnaA was prepared by incubation of apo-DnaA at 0 °C for 15 min in buffer N (50 mM Hepes-KOH [pH 7.6], 2.5 mM magnesium acetate, 0.3 mM EDTA, 7 mM dithiothreitol, 20% (v/v) glycerol, and 0.007% (v/v) Triton X-100) containing 3 μM [$^3$H]ADP, as described (1). The resultant [$^3$H]ADP-DnaA (2 pmol) was incubated in 25 μL of dissociation buffer (20 mM Tris-HCl [pH 7.5], 100 mM potassium glutamate, 10 mM magnesium acetate, 2 mM ATP, 8 mM dithiothreitol, and 100 μg/mL bovine serum albumin) containing 150 ng poly (dI-dC) and indicated amounts of *DARS2*, IHF and Fis. DnaA-bound [$^3$H]ADP were recovered on nitrocellulose filters, and analyzed as described (3).

In the reconstituted system of *DARS2* ATP-DnaA regeneration, apo-DnaA was incubated at 0 °C for 15 min in buffer N containing or excluding 3 μM ADP. The resultant apo- or ADP-DnaA (2 pmol) was further incubated in 25 μL of dissociation buffer which contained 1.5 μM

[α-$^{32}$P]ATP instead of 2 mM ATP, in addition to indicated amounts of *DARS2*, IHF and Fis. DnaA-bound [α-$^{32}$P]ATP was quantified as described above.

For the *DARS2*-RIDA-coupled reconstituted system, staged reactions were constructed. In the 1st reaction, ADP-DnaA (2 pmol) was incubated at 30 ˚C for 15 min in 25 μL of dissociation buffer containing 50 fmol each of IHF and Fis, 5 fmol of pOA61 and 1.5 μM [$^{32}$P]ATP, followed by further incubation for 5 min in the presence of PciI (0.4 U) for digestion of pOA61. In the 2nd reaction, indicated amounts of Hda and the DNA-loaded clamp (40 fmol as the clamp) were included in the 1 reaction, resulting in total volume of 28 μL, which was followed by further incubation at 30˚C for 20 min. DnaA-bound nucleotides were recovered on nitrocellulose filters, and analyzed by thin-layer chromatography, as previously described (9). The DNA-loaded clamp was prepared as described (9).

For the *DARS2*-DDAH-coupled reconstituted system, the 1st reaction including PciI digestion was performed as described above. In the 2nd reaction, indicated amounts of *datA* DNA and 0.2 pmol of IHF were included to the 1st reaction, resulting in the total volume of 28 μL, which was followed by further incubation for 10 min. DnaA-bound nucleotides were analyzed as described above.

In the *oriC* replication-reconstituted system coupled with *DARS2* ATP-DnaA regeneration, ADP- or ATP-DnaA (0.5 or 1 pmol , i.e., 20 or 40 nM) was incubated at 30 °C for 15 min in 25 μL of replication buffer (20 mM Tris-HCl [pH7.5], 125 mM potassium glutamate, 10 mM magnesium acetate, 8 mM dithiothreitol, 2 mM ATP, 1 mM each of GTP, CTP and UTP, 100 μM each of dNTP including [α-$^{32}$P]dATP, and 100 μg/mL bovine serum albumin) containing replication proteins (1.2 μM SSB, 20 nM clamp, 80 nM IHF, 80 nM GyrB, 130 nM His-GyrB, 20 nM DnaB, 22 nM DnaC, 100 nM DnaG, and 20 nM DNA polymerase III*), 5 fmol (0.2 nM) of pOA61 or pACYC177, and indicated amounts of Fis. Incorporation of [α-$^{32}$P]dAMP was quantified, as described (3, 5).

***Electrophoretic mobility shift assay***

For the experiments in Figure 3AB and Supplementary Figure S4, indicated amounts of IHF or Fis were incubated at 30 °C for 5 min in 12.5 μL of buffer GS (20 mM Hepes-KOH [pH 7.6], 50 mM potassium glutamate, 10 mM magnesium acetate, 1 mM EDTA, 8 mM dithiothreitol, 100 μg/mL bovine serum albumin, and 5% glycerol) containing 150 ng of poly (dI-dC) and indicated amounts of DNA, followed by analysis using 4%, 8%, or 10% PAGE at 100 V for 60–80 min in Tris-Borate buffer and staining with Gel-Star as described (6).

For the experiments in Figure 4B, *DARS2*ΔRight DNA (15 fmol) was incubated at 30 °C for 5 min in 8 μL of dissociation buffer containing 25 ng poly (dI-dC) and various amounts of wild-type ADP-DnaA or ADP-DnaA L290A, followed by analysis using 5% PAGE at 100 V for 100 min in Tris-Borate buffer and staining with GelStar.

For the experiments in Figure 4E and I, *DARS2*ΔRight/subFBS1 DNA (15 fmol) was incubated at 30 °C for 5 min in 8 μL of dissociation buffer containing 25 ng poly (dI-dC), 0.6 pmol

IHF, 0.3 pmol Fis and various amounts of ADP- or ATP-DnaA, followed by analysis using 5% PAGE at 100 V for 60–80 min in Tris-Borate buffer and phosphorimaging as described (3).

***Pull down assay***

Pull down experiments using *DARS2* were performed according to an *oriC* DNA pull down method which we previously described (10). [$^3$H]ADP-DnaA (2 pmol) was incubated at 30 °C for 5 min in 25 μL of dissociation buffer containing 2 mM ADP instead of ATP in addition to 0.5 pmol biotinylated *DARS2*ΔRight or *DARS2*ΔRightΔCore DNA and indicated amounts of IHF and Fis. *DARS2* and bound materials were recovered by Streptavidin MagneSphere Paramagnetic Particles (Promega), followed by SDS-10% PAGE, silver staining and quantification of DnaA.

**In vivo *DnaA-bound nucleotide analysis***

*In vivo* DnaA-immunoprecipitation was performed as described (3). Briefly, for the experiments in Figure 5C and G, cells were grown at 37 °C until the $A_{660}$ reached 0.1 in TG medium containing [$^{32}$P]orthophosphate, followed by immunoprecipitation using cell lysates and anti-DnaA antiserum. Recovered DnaA-bound nucleotides were analyzed by thin-layer chromatography and the ATP-DnaA level was calculated as ATP/(ATP+ADP) on DnaA [%].

For the experiments in Figure 5H, cells were grown at 28 °C until the $A_{660}$ reached 0.1, and then incubated at 42 °C for 20 min in the presence of 150 μg/mL chloramphenicol, followed by DnaA-immunoprecipitation and thin-layer chromatography as described above.

***Chromatin immunoprecipitation (ChIP)***

ChIP was performed according to a previously described method (6, 11), with minor modifications. In IHF- or Fis-ChIP experiments using synchronized cell cultures, KYA018 [*dnaC2*] cells were grown in supplemented M9 medium (15 mL) at 30 °C, a permissive temperature, until the $A_{660}$ reached 0.03, followed by further incubation at 38 °C, a restrictive temperature, for 90 min. Cells were then incubated at 30°C for 5 min, followed by further incubation at 30 °C or 38 °C for 5–45 min in the presence or absence of 300 μg/mL rifampicin. Samples were withdrawn at indicated time points. In Fis-ChIP experiments using non-synchronized cell cultures, MG1655 cells were grown in supplemented M9 medium at 38 °C until the $A_{660}$ reached 0.05, 0.2, 0.5, 1, or 2, or those cells were incubated overnight (the $A_{660}$ reached 2.6). Cells withdrawn were collected, and crosslinking and cell lysis were performed as described (6). Cell lysates were then sonicated six times for 20 sec each in IP buffer containing 50 mM Tris-HCl (pH 8.0), 250 mM NaCl, 1 mM EDTA, and 1% (vol/vol) Triton X-100 (6), followed by centrifugation at 14 krpm (16,000 × g) at 4 °C for 10 min. A portion (350 μL) of the supernatant was then mixed with 5 μL of polyclonal rabbit anti-IHF or anti-Fis antiserum and 60 μL of Protein A Sepharose 4 Fast Flow 50 % slurry (GE Healthcare), followed by incubation at 4 °C for 30 min with a gentle rotation. Beads and bound materials were washed sequentially, resuspended in 1% SDS, and incubated at 65 °C for 12 hr to allow de-crosslinking, as described (6). DNA in the samples before (Input) and after (ChIP)

immunoprecipitation was purified using a Wizard SV Gel and PCR Clean-Up System (Promega). The levels of *oriC*, *DARS2*, *ylcC*, and *ter* were quantified by real-time qPCR using SYBR Premix Ex Taq II (Perfect Real Time) (Takara Bio) and the following primers: ORI_1 and KWoriCRev for *oriC*, IHF-D2F and IHF-D2B for *DARS2*, RTYLCC-L and RTYLCC-R for *ylcC,* and TER_2 and SUEterRev1 for *ter* (see Supplementary Table S1 for each sequence).

The amounts of immunoprecipitated DNA were represented as ChIP values. As a quantitative standard, DNA in the cell lysate (5 μL) was similarly de-crosslinked, purified, and quantified by real-time qPCR as above; the resulting amounts were represented as the Input value. The *ylcC* region contains no specific IHF- and Fis-binding site and was used as a background control (12). The ratio of ChIP value to Input value (ChIP/Input) for *ylcC* was subtracted from the ChIP/Input value for *oriC,* and *DARS2* to calculate the value for specific IHF- or Fis-binding (6).

In addition, amounts of *ter* DNA in Input samples were quantified by real-time qPCR using the SUEterRev1 and TER_2 primers (Supplementary Table S1), and was used to calculate the *oriC*/*ter* and *DARS2*/*ter* ratios (6).

For Fis-ChIP experiments, denaturing conditions using 3M urea also were used as follows; after crosslinking, sonication and centrifugation described above, a portion (400 μL) of the supernatant was mixed with 200 μL of IP buffer containing 9 M urea and incubated at room temperature for 10 min with vigorous rotation, followed by dialysis against IP buffer at 4 °C overnight, accordingly to a method previously reported (13). The resulting sample was used for immunoprecipitation and DNA analysis as described above.

***Chromatin affinity precipitation (ChAP)***

ChAP was performed according to a previously described method (14), with minor modifications. In IHF-ChAP experiments using synchronized cell cultures, SH022 (*ihfA-cHis12 dnaC2*) cells were grown in supplemented M9 medium (5 mL) at 30 °C until the $A_{660}$ reached 0.03, followed by further incubation at 38 °C for 90 min. Cells were then incubated at 30 °C for 10–45 min. Samples were withdrawn at indicated time points. Crosslinking was performed as described (6), and the cells were then collected by centrifugation, washed twice with 1 mL of ice-cold TBS (50mM Tris-HCl [pH7.5] and 500 mM NaCl), resuspended in 500 μL of binding buffer (50 mM Tris-HCl [pH 7.5], 500 mM NaCl, 1% (vol/vol) Triton X-100, 5 mM imidazole, and complete Mini EDTA-free protease inhibitor mixture [Roche]), and sonicated six times for 20 sec each. The resulting size of the chromosomal DNA was about 1 kb. Cell debris was then removed by centrifugation at 14 krpm (16,000 × g) for 15 min at 4 °C, and a portion (400 μL) of the resulting supernatant (450 μL) was mixed with 10 μL of Dynabeads His-tag Isolation & Pulldown (lifetechnologies), followed by incubation at 4 °C for 1 hr with a gentle rotation. Beads and bound materials were washed four times with wash buffer (50 mM Tris-HCl [pH 7.5], 500 mM NaCl, 1% (vol/vol) Triton X-100, and 5 mM imidazole), resuspended in elution buffer (50 mM Tris-HCl [pH 7.5], 500 mM imidazole, 1% SDS, 10 mM EDTA, and 10 mM dithiothreitol), and incubated at 65 °C for 12 hr to allow de-crosslinking. DNA in the samples before (Input) and after (ChAP) pull

down was purified using a Wizard SV Gel and PCR Clean-Up System (Promega). The levels of *DARS2* and *ylcC* were quantified by real-time qPCR (6).

**Supplementary References**


1. Nishida, S., Fujimitsu, K., Sekimizu, K., Ohmura, T., Ueda, T. and Katayama, T. (2002) A nucleotide switch in the *Escherichia coli* DnaA protein initiates chromosomal replication: evidnece from a mutant DnaA protein defective in regulatory ATP hydrolysis *in vitro* and *in vivo*. *J. Biol. Chem.*, **277**, 14986–14995.
2. Kawakami, H., Ozaki, S., Suzuki, S., Nakamura, K., Senriuchi, T., Su'etsugu, M., Fujimitsu, K. and Katayama, T. (2006) The exceptionally tight affinity of DnaA for ATP/ADP requires a unique aspartic acid residue in the AAA+ sensor 1 motif. *Mol. Microbiol.*, **62**, 1310–1324.
3. Fujimitsu, K., Senriuchi, T. and Katayama, T. (2009) Specific genomic sequences of *E. coli* promote replicational initiation by directly reactivating ADP-DnaA. *Genes Dev.*, **23**, 1221–33.
4. Filutowicz, M., Grimek, H. and Appelt, K. (1994) Purification of the *Escherichia coli* integration host factor (IHF) in one chromatographic step. *Gene*, **147**, 149–150.
5. Ozaki, S., Noguchi, Y., Hayashi, Y. and Katayama, T. (2012) Differentiation of the DnaA-*oriC* Subcomplex for DNA Unwinding in a Replication Initiation Complex. *J. Biol. Chem.*, **287**, 37458–37471.
6. Kasho, K. and Katayama, T. (2013) DnaA binding locus *datA* promotes DnaA-ATP hydrolysis to enable cell cycle-coordinated replication initiation. *Proc. Natl. Acad. Sci. U. S. A.*, **110**, 936–941.
7. Datsenko, K.A. and Wanner, B.L. (2000) One-step inactivation of chromosomal genes in *Escherichia coli* K-12 using PCR products. *Proc. Natl. Acad. Sci. U. S. A.*, **97**, 6640–645.
8. Hatano, T., Yamaichi, Y. and Niki, H. (2007) Oscillating focus of SopA associated with filamentous structure guides partitioning of F plasmid. *Mol. Microbiol.*, **64**, 1198–1213.
9. Nakamura, K. and Katayama, T. (2010) Novel essential residues of Hda for interaction with DnaA in the regulatory inactivation of DnaA: unique roles for Hda AAA Box VI and VII motifs. *Mol. Microbiol.*, **76**, 302–317.
10. Keyamura, K., Abe, Y., Higashi, M., Ueda, T. and Katayama, T. (2009) DiaA Dynamics Are Coupled with Changes in Initial Origin Complexes Leading to Helicase Loading. *J. Biol. Chem.*, **284**, 25038–25050.
11. Cho, B., Knight, E.M., Barrett, C.L. and Palsson, B.Ø. (2008) Genome-wide analysis of Fis binding in *Escherichia coli* indicates a causative role for A-/AT-tracts. *Genome Res.*, **18**, 900–910.
12. Grainger, D.C., Hurd, D., Goldberg, M.D. and Busby, S.J.W. (2006) Association of nucleoid proteins with coding and non-coding segments of the *Escherichia coli* genome. *Nucleic Acids Res.*, **34**, 4642–4652.
13. Boehm, A.K., Saunders, A., Werner, J., Lis, T., Boehm, A.K., Saunders, A., Werner, J. and Lis, J.T. (2003) Transcription Factor and Polymerase Recruitment, Modification, and Movement on *dhsp70* In Vivo in the Minutes following Heat Shock. *Mol. Cell. Biol.*, **23**, 7628–7637.

14. Su'etsugu, M. and Errington, J. (2011) The replicase sliding clamp dynamically accumulates behind progressing replication forks in *Bacillus subtilis* cells. *Mol. Cell*, **41**, 720–732.
15. Aeling, K.A., Opel, M.L., Steffen, N.R., Tretyachenko-Ladokhina, V., Hatfield, G.W., Lathrop, R.H. and Senear, D.F. (2006) Indirect recognition in sequence-specific DNA binding by *Escherichia coli* integration host factor: the role of DNA deformation energy. *J. Biol. Chem.*, **281**, 39236–39248.
16. Stella, S., Cascio, D. and Johnson, R.C. (2010) The shape of the DNA minor groove directs binding by the DNA-bending protein Fis. *Genes Dev.*, **24**, 814–826.
17. Fujimitsu, K., Su'etsugu, M., Yamaguchi, Y., Mazda, K., Fu, N., Kawakami, H. and Katayama, T. (2008) Modes of Overinitiation, dnaA Gene Expression, and Inhibition of Cell Division in a Novel Cold-Sensitive *hda* Mutant of *Escherichia coli*. *J. Bacteriol.*, **190**, 5368–5381.
18. Kurokawa, K., Nishida, S., Emoto, a, Sekimizu, K. and Katayama, T. (1999) Replication cycle-coordinated change of the adenine nucleotide-bound forms of DnaA protein in *Escherichia coli*. *EMBO J.*, **18**, 6642–6652.
19. Kato, J. and Katayama, T. (2001) Hda, a novel DnaA-related protein, regulates the replication cycle in *Escherichia coli*. *EMBO J.*, **20**, 4253–4262.

## Supplementary Figure Legends

**Supplementary Figure S1.** Right half of *DARS2* and DnaA boxes IV-VI are dispensable for initiation stimulation.

(A) Analysis of truncated *DARS2* derivatives. MG1655 (WT) cells bearing pACYC177 (vector), pOA61 (*DARS2* WT [wild-type]), pOA71 (ΔCore), pOA67 (ΔRight), pOA73 (Δ(Right+21)), or pOA86 (Δ21) were grown at 37 °C in supplemented M9-ampicillin medium, followed by incubation in the presence of rifampicin and cephalexin. The upper and lower numbers inserted in histograms indicate relative ratios of cell mass and ori/mass compared with those of cells bearing pACYC177.

(B) Analysis of *DARS2* DnaA box mutants. MG1655 cells bearing pACYC177, pOA61, pKX44 (subDnaAboxIV), pKX45 (subDnaAboxV), or pKX46 (subDnaAboxVI) were similarly analyzed by flow cytometry. In pKX44, pKX45, and pKX46, DnaA box IV, DnaA box V and DnaA box VI in *DARS2* were respectively substituted with a non-specific sequence that is impaired in DnaA binding (6).

**Supplementary Figure S2.** Analysis of IHF, Fis and HU in *DARS2*-medicated ADP dissociation.

(A) Quantification of IHF in a crude extract. Proteins in 10 μg of YH014 (WT) Fr II and in mixtures of 10 μg of YH014-I (*ihfA*::Tn*10*) Fr II and the indicated amounts of purified IHF were separated by SDS-20% PAGE and analyzed by western blotting using anti-IHF antiserum. Band intensities were quantified, background intensity was subtracted, and the amounts of IHF in YH014 Fr II were deduced. Two independent experiments were performed and the mean value with the different ranges is shown for the IHF included.

(B) Quantification of Fis in a crude extract. Proteins in 10 μg of MG1655 (WT) Fr II and in mixtures of 10 μg of KMG-2 (Δ*fis*) Fr II and the indicated amounts of purified Fis were separated by SDS-20% PAGE and analyzed by western blotting using anti-Fis antiserum. Band intensities were quantified and the amounts of Fis in MG1655 Fr II were deduced. Two independent experiments were performed and the mean value with the different ranges is shown for the Fis included. *, non-specific signal.

(C) Analysis using a reconstituted system. [$^3$H]ADP-DnaA (2 pmol) was incubated at 30 °C for 15 min with 5 fmol pOA61 and indicated amounts of HU (▲, △) or IHF (●,○) in the presence (●, ▲) or absence (○, △) of 50 fmol Fis. Two independent experiments were done for each assay, and both data and mean values are shown.

(D) Analysis using a crude protein extract. Titration of HU in the presence or absence (○) of 50 fmol each of IHF (▲, ■) and Fis (▲), or 10 μg MG1655 crude protein extract (Fr II) (●). Two independent experiments were done for each assay, and both data and mean values are shown.

**Supplementary Figure S3.** Sequence analysis of *DARS2* footprint and determination of IHF- and Fis-binding sites.

(A) Structure of *DARS2* including IBS and FBS determined in Figure 3. Black or dotted arrowheads represent DnaA boxes that are completely identical with the 9-mer consensus sequence (DnaA boxes I–VI) or that contain only a single mismatch (DnaA boxes I–VI), respectively. Green or blue lines represent IHF- or Fis-binding sites, i.e., IBS1-2, FBS1, FBS2-5 and FBS6, respectively, which were determined in Figure 3.
(B) Sequences of IBS. Based on the results shown in Figure 3C, the sites protected or hypersensitive to DNase I in the presence of 1.2 pmol of IHF are indicated by x or l, respectively. Green letters and boxes indicate the IHF-binding consensus. Sequences identical to the consensus are indicated by asterisks.
(C) Sequences of FBS. Based on the results shown in Figure 3D, the sites protected or hypersensitive to DNase I in the presence of Fis are indicated as described for panel A. A set of Upper-1 and Lower-1 indicates the results in the presence 0.12 pmol of Fis, and a set of Upper-2 and Lower-2 indicates those in the presence 1.9 pmol of Fis. Blue letters and boxes indicate the Fis-binding consensus. Sequences identical to the consensus are indicated by asterisks.

**Supplementary Figure S4.** Construction of IBS and FBS mutants.
(A) Schematic view of mutations introduced to IBS and FBS. The native sequences which are identical to the IBS or FBS consensus are indicated by asterisks (15, 16). Substituted bases are displayed by red letters.
(B) Site-specific binding of IHF. The indicated amounts of IHF were incubated at 30 °C for 5 min in buffer GS containing 0.1 pmol of *DARS2* WT or subIBS1-2 (455 bp) in the presence 150 ng poly (dI-dC), followed by 4% PAGE. Well: gel well, Bound: protein-bound DNA, Free: protein-free DNA.
(C-F) Site-specific binding of Fis. The indicated amounts of Fis were similarly incubated with 0.3 pmol of each DNA at 30 °C for 5 min, followed by 8% PAGE (C, F) or 10 % PAGE (D, E). DNA used were: FBS1 and subFBS1 (C), FBS2-3 and subFBS2-3 (D), FBS4-5 and subFBS4-5 (E), and FBS6 and subFBS6 (F).

**Supplementary Figure S5.** FBS1 and FBS6 are dispensable for *DARS2* activation *in vitro*.
(A) Analysis using a crude protein extract. [$^3$H]ADP-DnaA (2 pmol) was incubated at 30 °C for 15 min with indicated amounts of pOA61 (*DARS2* WT) (●), pKX25 (subFBS1) (▲), pKX28 (subFBS6) (◆), and pACYC177 vector (None)] (○) in the presence of MG1655 Fr II (10 μg).
(B) Analysis using reconstituted reactions. Similar experiments were performed in the presence of 50 fmol each of IHF and Fis.

**Supplementary Figure S6.** Analysis of translocation of IBS1-2 and FBS2-3 in *DARS2* activation.
(A) Sequences of IBS1-2 and FBS2-3 translocated. The nucleotide number is identical to that used in Figure 1D. The location of IBS1-2 or FBS2-3 was translocated by 5 bp or 10 bp. The IBS1-2– or

FBS2-3–flanking 5-bp or 10-bp sequence was moved from the left side to the right side or *vice versa*.

(B) Analysis using *in vitro* ADP dissociation reconstituted system. [$^3$H]ADP-DnaA (2 pmol) was incubated with 5 fmol of pOA61 (WT), pKX98 (traIBS-L5), pKX99 (traIBS-L10), pKX100 (traIBS-R5), pKX101 (traIBS-R10), pKX102 (traFBS-L5), pKX102 (traFBS-L10), or pACYC177 vector (None), under the conditions same as those used for experiments of Figure 2C. Two independent experiments were done for each assay, and both data and mean values are shown.

**Supplementary Figure S7.** ADP dissociation of DnaA sensor I mutant D269N.

(A) *DARS2*-mediated ADP dissociation from wild-type DnaA and DnaA D269N. [$^3$H]ADP-DnaA or [$^3$H]ADP-DnaA B269N (2 pmol) was incubated at 30 °C for 15 min with 50 fmol each of IHF and Fis, and indicated amounts of pOA61 (*DARS2* WT) (+) or pACYC177 vector (–) in the presence (+) or absence (–) of 150 ng poly (dI-dC). Two independent experiments were done for each assay, and both data and mean values are shown.

(B) *DARS1*-mediated ADP dissociation from wild-type DnaA and DnaA D269N. [$^3$H]ADP-DnaA or [$^3$H]ADP-DnaA B269N (2 pmol) was incubated at 30 °C for 15 min with indicated amounts of FK7-7 (*DARS1* WT) (+) or FK7-21 (ΔCore) (–) in the presence (+) or absence (–) of 150 ng poly (dI-dC). Two independent experiments were done for each assay, and both data and mean values are shown.

**Supplementary Figure S8.** Specific recovery of DnaA-bound nucleotides in immunoprecipitation. KW262-5 cells were grown at 37 °C until the $A_{660}$ reached 0.1 in TG medium containing [$^{32}$P]orthophosphate, followed by immunoprecipitation using cell lysates and pre-immune serum or anti-DnaA antiserum. Recovered materials were analyzed by thin-layer chromatography and phosphorimaging (17, 18). The origin of the chromatography and DnaA-bound nucleotides are indicated (Origin, ATP and ADP).

**Supplementary Figure S9.** Regulations of binding of IHF and Fis to *DARS2*.

(A) IHF-ChIP on *oriC*. KYA018 (*dnaC2*) cells were incubated as described for Figure 6A. The ChIP/Input [%] for *oriC* was deduced using a control *ylcC* as described in Supplementary Materials and Methods. Error bars represent the standard deviation from at least two independent experiments.

(B and C) IHF-ChAP on *oriC* and *DARS2*. SH022 (*ihfA-12His dnaC2*) cells growing at 30 °C were transferred to 38 °C and further incubated for 90 min. Cells were then transferred to 30 °C (Time 0) and further incubated for 20 min. The relative levels of *oriC* (B) and *DARS2* (C) before (Input) and after (IHF-ChAP) recovery using cobalt-conjugated beads were determined using real-time qPCR, yielding the ChAP/Input ratio [expressed as %], as described for Figure 6A and B. Two independent experiments were done, and both data and mean points are shown.

(D) IHF-ChIP on *DARS2* in the presence of rifampicin. KYA018 cells were incubated as described

for Figure 7A except that 300 $\mu$g/mL rifampicin was included at the time of temperature shift to 30 °C (Time 0). Analyses of ChIP for IHF binding and *DARS2*/*ter* ratios also were performed similarly. Error bars represent the standard deviation from at least two independent experiments.
(E) Fis-ChIP on *DARS2*. KYA018 cells were incubated as described for Figure 6B, and the *DARS2*-specific Fis-ChIP/Input [%] and *DARS2*/*ter* ratios were determined as described for Figure 7A.
(F) Fis-ChIP using potent denaturing conditions. MG1655 cells were incubated at 30 °C or at 38 °C, and samples were withdrawn as described for Figure 6C. ChIP experiments were performed including a step for exposure to 3M urea, as described in Supplementary Materials and Methods.
(G) IHF-ChIP on *DARS2* at stationary phase. MG1655 cells were incubated at 38 °C in supplemented M9 medium until the indicated $A_{660}$ or overnight (O/N, $A_{660}$ of 2.6). The *DARS2*-specific IHF-ChIP/Input [%] was determined as described for Figure 7A.

**Supplementary Figure S10.** Conservation and roles for the *DARS2* core, IBS1-2 and FBS2-3.
(A) Homology of sequences corresponding to the *E. coli DARS2* core, IBS1-2 and FBS2-3 in the genomes of *E. coli*-proximal 8 bacterial species were analyzed using the Blast search tool and the NCBI database. Arrows indicate the direction of the DnaA box. Sequences that are identical with the consensus DnaA box (TTATnCACA), IBS (TAAnnnnTTGATW, where W is A or T), and FBS (GnnYAnnnnnTRnnC, where Y is T or C and R is A or G) are highlighted. Eco, *Escherichia coli K-12*; Sfl, *Shigella flexneri 2a 2457T*; Eca, *Erwinia carotovora atroseptica SCRI1043*; Sty, *Salmonella typhimurium LT2*; Ype, *Yersinia peptis KIM*; Vch, *Vibrio cholerae El Tor N16961*; Plu, *Photorhabdus luminescens TTO1*; Sde, *Shewanella denitrificans OS217*; Ahy, *Aeromonas hydrophila subsp. Hydrophila ATCC 7966*.
(B) Mechanistic model of *DARS2* DnaA-ADP dissociation. IHF and Fis bind to specific sites (IBS1-2 and FBS2-3, respectively), and the resultant IHF-Fis bound DNA region stimulates conformational change of the core DnaA boxes (Core)-bound DnaA complex, which causes specific structural change in DnaA protomers, resulting in ADP dissociation. The resultant apo-DnaA binds ATP, yielding ATP-DnaA. For simplicity, only single molecules of IHF and Fis are shown in this figure.

**Supplementary Table S1. List of oligonucleotides**

| Primers | Sequences |
|---|---|
| Fis-1 | GGAATTCCATATGTTCGAACAACGCGTAAATTCTG |
| Fis-3 | CCGCTCGAGGTTCATGCCGTATTTTTTC |
| MutH-4 | CCCAAGCTTGAGGAAGGGGTGGATAGCC |
| MutH-11 | CGGGATCCTACGGCATTGGTTGATCTTTC |
| MutH-17 | CGGGATCCGCCAACCCGTAAATGAGAGG |
| MutH-14 | AAACGGCTATCCACCCCTTCC |
| MutH-15 | CTACGGAATTACTACGGGAAAAC |
| MutH-24 | CGCCAACCCGTAAATGAGAGG |
| MutH-25 | TTATCCACAGAATGTGCCACTAAG |
| Ksh-16 | TGTACTTTATTTTAAAATGTCTATATCGGGC |
| Ksh-19 | TCGTACTTTGCATTACATGTAATTTTAGTAATG |
| Ksh-3 | ACATATGTTTTTCATTACTAAAATTACATGTAATGCATTG |
| Ksh-4 | TCGATACGCAGGTCACACCTCTCATTTACGGG |
| Ksh-20 | TCTTATGCGAACCCGTAAATGAGAGGTGTGAC |
| Ksh-21 | TCTACGAAAGCCGTATTTATCCACAGAATGTG |
| Ksh-22 | TGAATAAATATGGCATTGGTTGATCTTTCGC |
| Ksh-23 | CAGACATATGCACTAAGTTAAGCACTGAACCACTAAAAAC |
| Ksh-9 | TGTATCGGAACTCGATTACCGGCAACCTAAAAAGC |
| Ksh-10 | CCATATGGGGTTTTCCAAATCTGGTCACTG |
| Ksh-30 | TATTGCCCGATATAGACATTTTAAAATATTC |
| Ksh-31 | CATGTATGCTCCGGGTTTTCCC |
| Ksh-32 | GACTGAATGTGCCACTAAGTTAAGCAC |
| Ksh-33 | CATGTATACGGCATTGGTTGATCTTTC |
| Ksh-34 | TATTCATGGTGTAAGATCCTGTTTATTTTC |
| Ksh-35 | CATGTGAAAGCTGGGATAACTGTGAAAAAC |
| traIL-U | CATGTAATTTTAGTAATGAAAAAGAGTAATTC |
| traIL-L1 | AAATGTAATGCATTGACTGATAATGAATATTTTATCTATATCGGGCTTA TTCAGAATG |
| traIL-L2 | AAATGTCTATTAATGCATTGACTGATAATGAATATTTTAATCGGGCTTA TTCAGAATGC |
| traIR-U1 | CATGTTAAAATATTCATTATCAGTCAATGCATTAAATTTTAGTAATGAA AAAGAGTAATTCGTG |
| traIR-U2 | CATGTAATTTTAAAATATTCATTATCAGTCAATGCATTATAGTAATGAA AAAGAGTAATTCGTGACC |
| traIR-L | AAATGTCTATATCGGGCTTATTCAG |

| | |
|---|---|
| traF-U | TATTTATCCACAGAATGTGCCACTAAG |
| traF-L1 | AACCCCGGCATTGGTTGATCTTTCGCCGTAAATGAGAGGTGTGACCTGGG |
| traF-L2 | AACCCGTAAACGGCATTGGTTGATCTTTCGCCTGAGAGGTGTGACCTGGGTCAC |
| insIBS-10U | TAAAATATTCATTATCAGTCAATGCATTAAGGTCACACCTCTCATTTACGGG |
| insIBS-L | AAATTACATGTAATGCATTGACTGATAATG |
| MutH-2 | CGGGATCCATTGCTTTTTAGGTTGCCG |
| pOA61-FP1 | GAATAACGGTTTGGTTGATGCG |
| pOA61-FP2 | CGGTACGCCTGCGGCC |
| FIS-1U | ATGAAAAAGAGTAATTCGTGACCCAGGTCAC |
| FIS-1L | GTGACCTGGGTCACGAATTACTCTTTTTCAT |
| FISsub-5U | ATGAAAAACATATGTTCGATACGCAGGTCAC |
| FISsub-5L | GTGACCTGCGTATCGAACATATGTTTTTCAT |
| F23U | TACGGGTTGGCGAAAGATCAACCAATGCCGTATTTATC |
| F23L | GATAAATACGGCATTGGTTGATCTTTCGCCAACCCGTA |
| subF23U | TACGGGTTCGCATAAGATCTACGAAAGCCGTATTTATC |
| subF23L | GATAAATACGGCTTTCGTAGATCTTATGCGAACCCGTA |
| F45U | CAACCAATGCCGTATTTATCCACAGAATGTGCCACTAAGT |
| F45L | ACTTAGTGGCACATTCTGTGGATAAATACGGCATTGGTTG |
| subF45U | CAACCAATGCCATATTTATTCACAGACATATGCACTAAGT |
| subF45L | ACTTAGTGCATATGTCTGTGAATAAATATGGCATTGGTTG |
| FIS-4U | GAAAACCCGTTGCAGTGTTGCGCAACTCGAT |
| FIS-4L | ATCGAGTTGCGCAACACTGCAACGGGTTTTC |
| FISsub-4U | GAAAACCCCTTAAGGTGTTGCGCAACTCGAT |
| FISsub-4L | ATCGAGTTGCGCAACACCTTAAGGGGTTTTC |
| Ksh-26 | AAAAAGCACCCGACAGGTGCTTTTCTCTCGTTCAAGTTTGAGTAAAAAACACCTGATAGTTTGGCTGTG |
| Ksh-27 | AATGCAGCAACAGCAGCCGCTTAATTTGCCTTTAAGGAACCGGAGGAATCGAAGCCAGGGCAGATCCG |
| D1spec-U | AATGTGGGAATTGCCCAGGCGGCGGGGGATAGGGGCTGGAGACAGACCTGATAGTTTGGCTGTG |
| D1spec-L | CGTGCAAGCCGCGTATTCTCTCGCTTGCCTCGTGTTTTCTAACTCGAAGCCAGGGCAGATCCG |
| TOP1829 | CCTTCAGACCCGGGCAGAAGTTAAAAAGCCGGGTCGAAAACGCTTCGCCCAAAGACGAGAGAGGATCGCATCACCATCACCATC |
| TOP1830 | ACGGTGACTCTTCGACAGTGAAAAGAAAAAAGGCCGCAGAGCGGCCTT |

| | |
|---|---|
| | TTTAGTTAGATCCATATGAATATCCTCCTCCTTAG |
| D2TET-1 | CACTAATAACAATTGAATAACTCACAGTTATGTGCAGAGTTATAAACAGA |
| | AATTCTCATGTTTGACAGC |
| MutH-2Nosite | ATTGCTTTTTAGGTTGCCG |
| Ksh-14 | TCTATCCACAGAAAAGGTGAATAAAAACGGCTATCCACCCCTTCCTCAG |
| | TGTAGGCTGGAGCTGCTTC |
| Ksh-15 | CACTAATAACAATTGAATAACTCACAGTTATGTGCAGAGTTATAAACGTC |
| | CATATGAATATCCTCCTTAG |
| ORI_1 | CTGTGAATGATCGGTGATC |
| KWoriCRev | GTGGATAACTCTGTCAGGAAGCTTG |
| IHF-D2F | GTCACACCTCTCATTTACGGG |
| IHF-D2B | CCAGTTTTTAGTGGTTCAGTGC |
| RTYLCC-L | GGCGTGGTAAAGGGTATCG |
| RTYLCC-R | TCTGCGGGGTGATGGTAAAG |
| TER_2 | TATCTTCCTGCTCAACGGTC |
| SUEterRev1 | GAACTACGCGGGAAATACC |

**Supplementary Table S2. List of *E. coli* strains**

| Strain | Relevant genotype | Source |
|---|---|---|
| BL21(λDE3) | *ompT gal dcm lon* $hsdS_B$ and λ prophage DE3 carrying T7 RNA polymerase | Laboratory stock |
| MG1655 | Wild type | Laboratory stock |
| YH014 | MG1655 *hda*+ *kan* | (17) |
| YH014-I | YH014 *ihfA*::Tn*10* | This work |
| KMG-5 | MG1655 Δ*ihfA*::*frt-kan* | This work |
| KMG-6 | MG1655 Δ*ihfB*::*frt-kan* | This work |
| KMG-2 | MG1655 Δ*fis*::*frt-kan* | This work |
| KP245 | *thyA trp his metB lac gal tsx* | (18) |
| KP7364 | KP245 Δ*dnaA*::*spec rnhA*::*kan* | (18) |
| KW262-5 | MG1655 *rnhA*::Tn*3 oriC* del-1071::Tn*10* | (19) |
| MK86 | KW262-5 Δ*hda*::*cat* | (19) |
| MIT47 | MK86 *DARS1*ΔCore::*kan* | (3) |
| MIT86 | MK86 *DARS2*ΔCore::*spec* | (3) |
| KX95 | MG1655 Δ*ihfB*::*spec* | This work |
| KX97 | MK86 Δ*ihfB*::*spec* | This work |
| KX101 | KX97 *DARS1*ΔCore::*kan* | This work |
| KX31 | MK86 Δ*ihfB*::*kan* | (6) |
| KX90 | KX31 *DARS2*ΔCore::*spec* | This work |
| KX93 | MK86 Δ*datA*::*kan* | (6) |
| KX176 | MG1655 *DARS1*ΔCore::*spec* | This work |
| KX179 | KX93 *DARS1*ΔCore::*spec* | This work |
| KX102 | KX93 *DARS2*ΔCore::*spec* | This work |
| KX29 | MK86 Δ*fis*::*frt-kan* | This work |
| KA474 | *dnaN59 tnaA*::*Tn*10 | (18) |
| MIT78 | MG1655 *DARS2*ΔCore::*cat* | (3) |
| MIT187 | MG1655 *DARS2* WT-*tet* | This work |
| KX53 | MG1655 *DARS2*subIBS1–2-*tet* | This work |
| KX5 | MG1655 *DARS2*subFBS1-*tet* | This work |
| KX54 | MG1655 *DARS2*subFBS2–3-*tet* | This work |
| KX55 | MG1655 *DARS2*subFBS4–5-*tet* | This work |
| KX7 | MG1655 *DARS2*sub6-*tet* | This work |
| MIT17 | MG1655 *DARS1*ΔCore::*kan* | (3) |
| MIT80 | MIT17 *DARS2*ΔCore::*cat* | (3) |
| KX58 | MIT17 *DARS2*subIBS1–2-*tet* | This work |

| | | |
|---|---|---|
| KX8 | MIT17 *DARS2*subFBS1-*tet* | This work |
| KX59 | MIT17 *DARS2*subFBS2–3-*tet* | This work |
| KX60 | MIT17 *DARS2*subFBS4–5-*tet* | This work |
| KX10 | MIT17 *DARS2*subFBS6-*tet* | This work |
| KX41 | MK86 *DARS2* WT-*frt-kan* | This work |
| KX68 | MK86 *DARS2*subIBS1–2-*frt-kan* | This work |
| KX69 | MK86 *DARS2*subFBS2–3-*frt-kan* | This work |
| KX70 | MK86 *DARS2*subFBS4–5-*frt-kan* | This work |
| KYA018 | MG1655 *dnaC2 zjj18*::*cat* | (6) |
| BW25113 | *rrnB* DE*lacZ*4787 *HsdR*514 DE(*araBAD*)567 DE(*rhaBAD*)568 *rph*-1 | (7) |
| SH022 | MG1655 *ihfA-cHis12 dnaC2 zjj18*::*cat* | This work |

**Supplementary Figure S1**

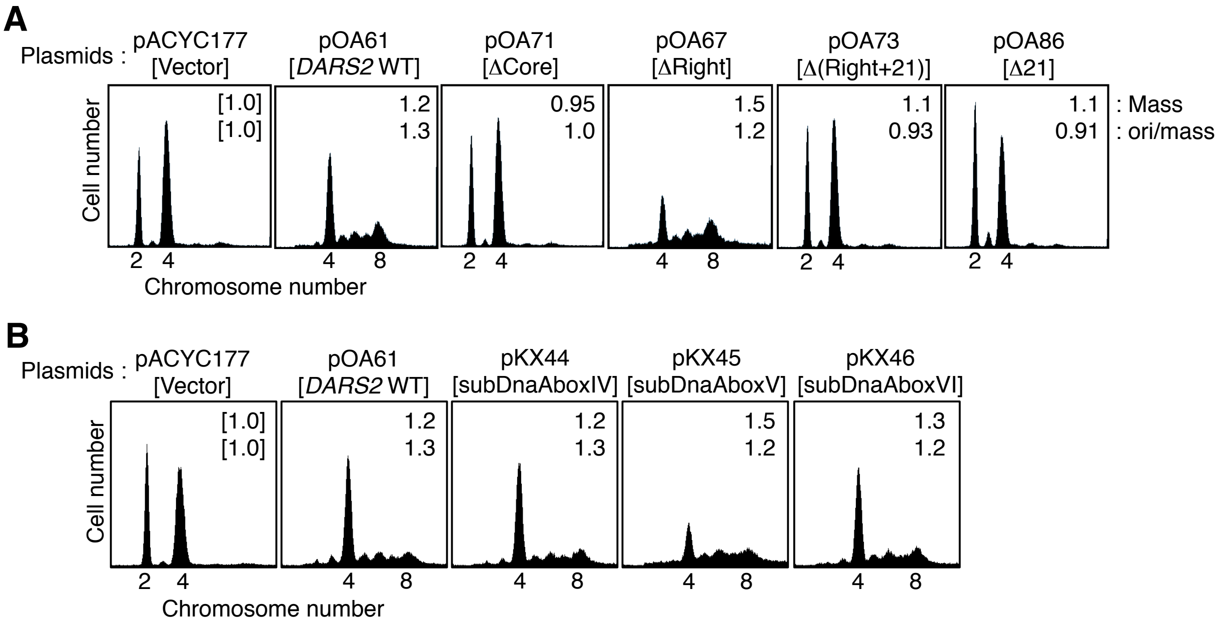

## Supplementary Figure S2

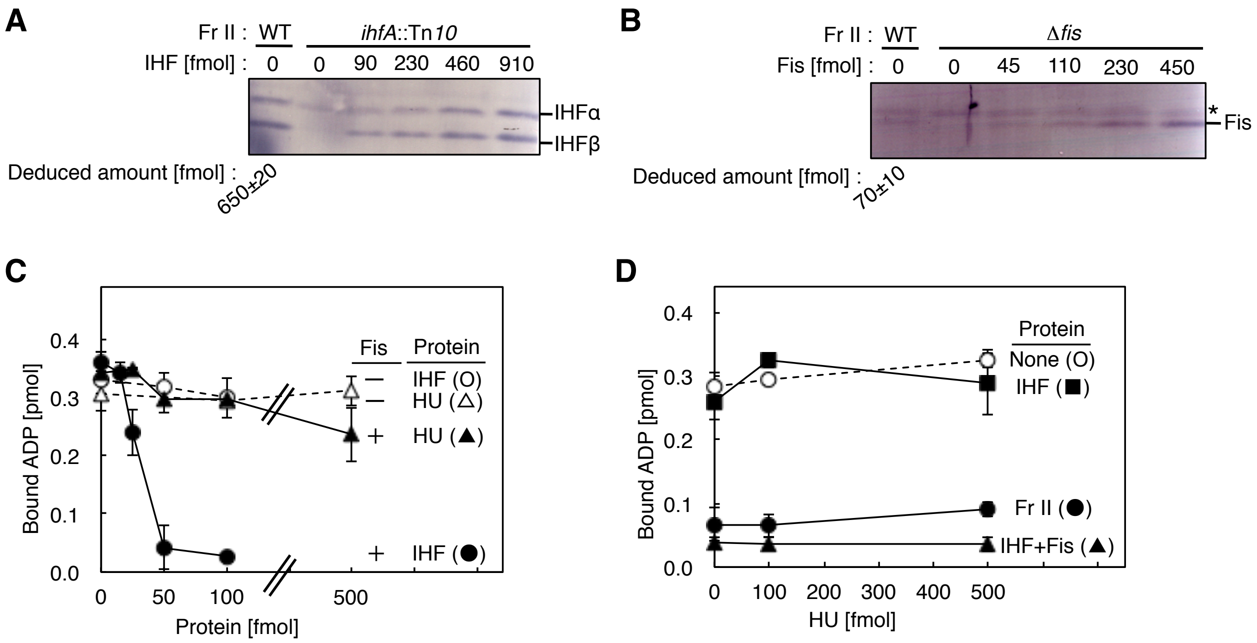

**Supplementary Figure S3**

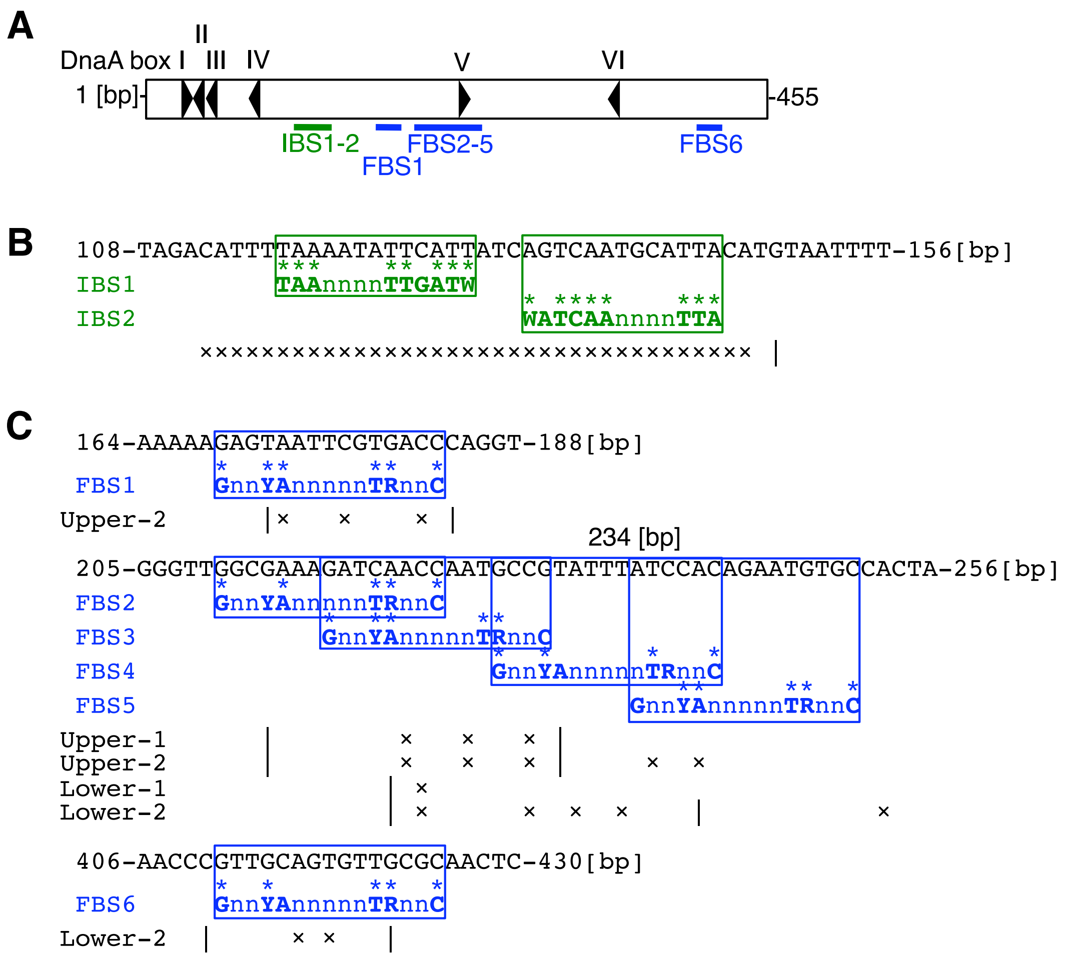

# Supplementary Figure S4

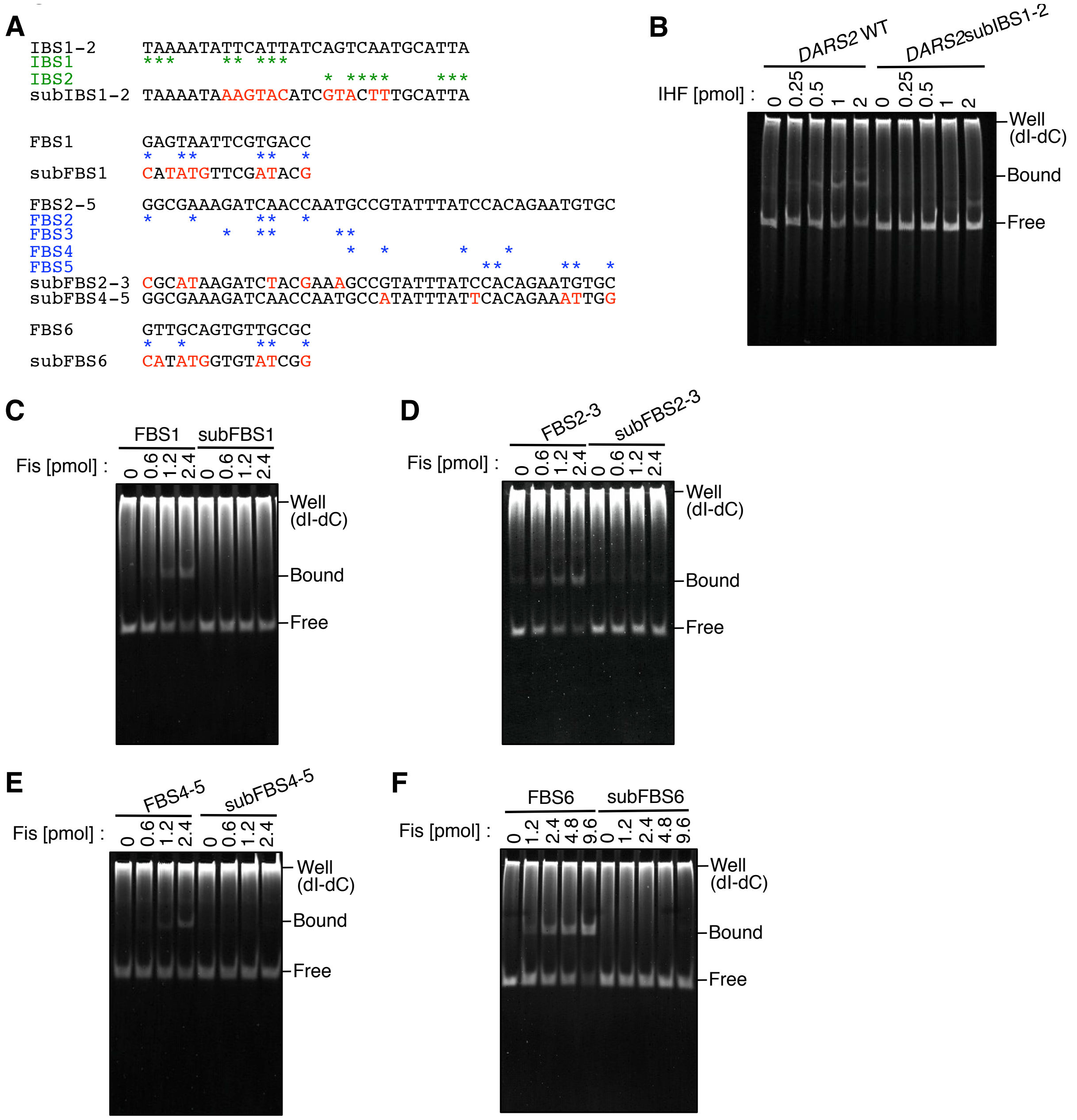

**Supplementary Figure S5**

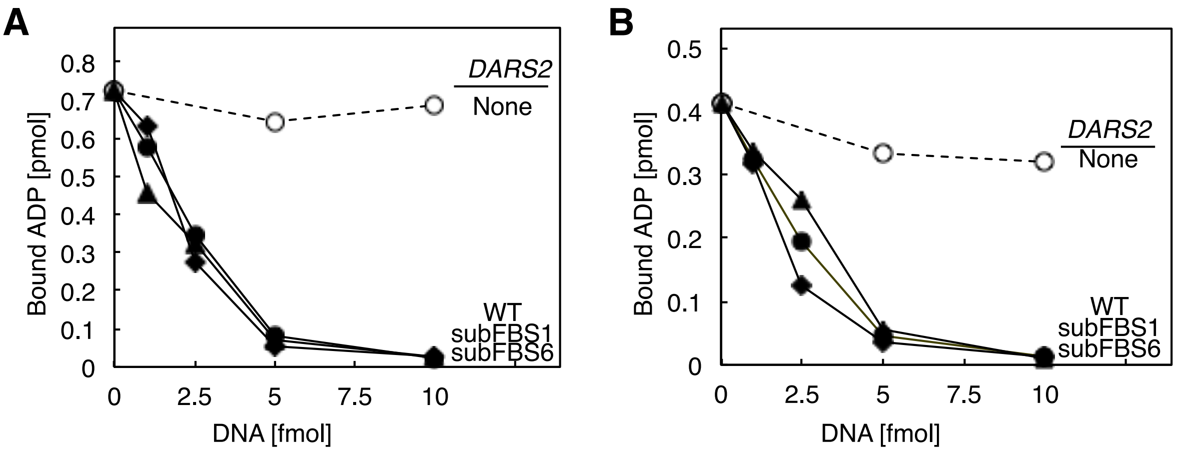

## Supplementary Figure S6

**A**

```
                                    104                                                          158 [bp]
WT          (Core)...GATATAGACATTTTAAAATATTCATTATCAGTCAATGCATTACATGTAATTTTAG...(FBS2-3)
                                                IBS1-2
traIBS-R5            GATATAGACATTTCATGTTAAAATATTCATTATCAGTCAATGCATTAAATTTTAG
traIBS-R10           GATATAGACATTTCATGTAATTTTAAAATATTCATTATCAGTCAATGCATTATAG
traIBS-L5            GATATAGATAAAATATTCATTATCAGTCAATGCATTACATTTCATGTAATTTTAG
traIBS-L10           GATTAAAATATTCATTATCAGTCAATGCATTAATAGACATTTCATGTAATTTTAG

                     197                                    234 [bp]
WT        (IBS1-2)...TCATTTACGGGTTGGCGAAAGATCAACCAATGCCGTAT...(DnaA box V)
                                          FBS2-3
traFBS-L5            TCATTTACGGCGAAAGATCAACCAATGCCGGGGTTTAT
traFBS-L10           TCAGGCGAAAGATCAACCAATGCCGTTTACGGGTTTAT
```

**B**

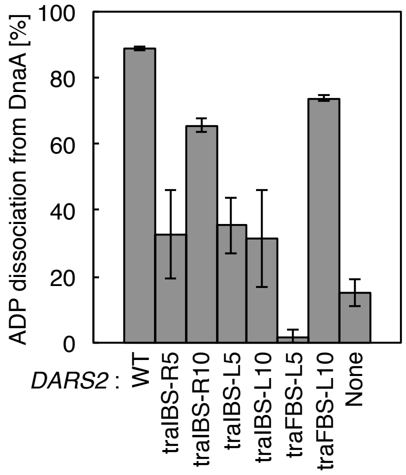

## Supplementary Figure S7

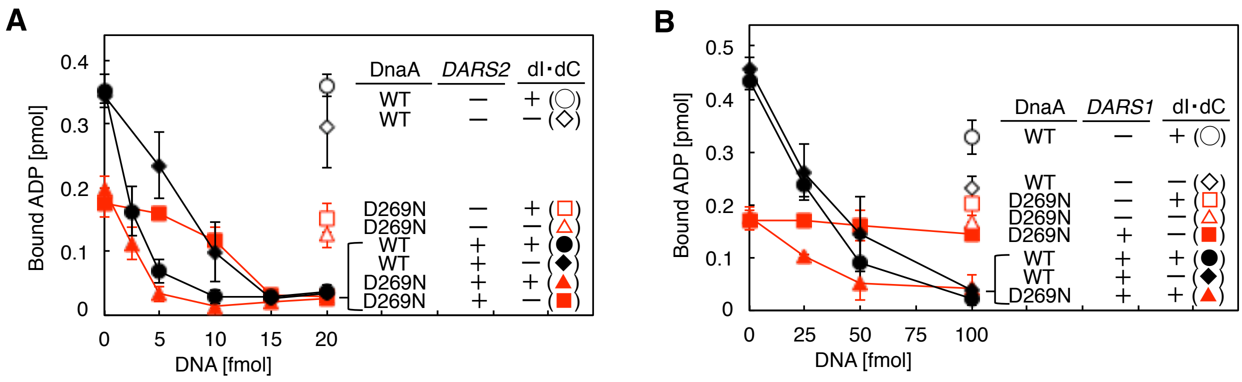

**Supplementary Figure S8**

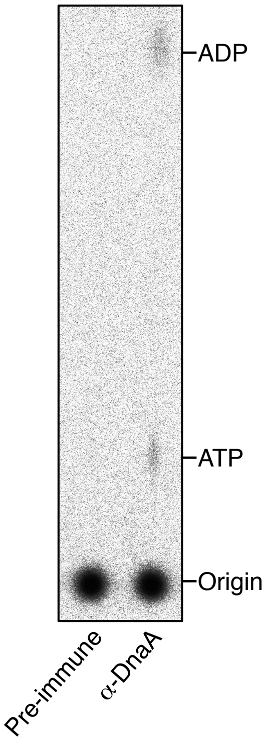

## Supplementary Figure S9

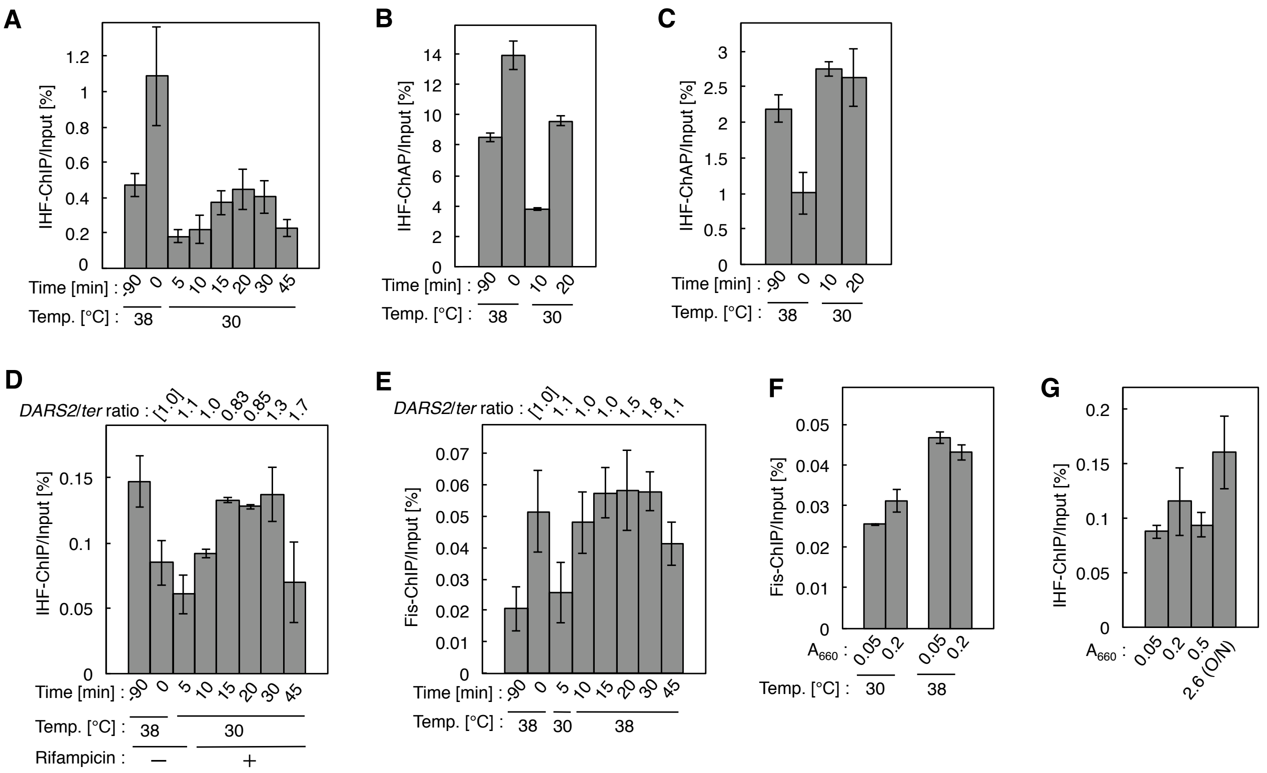

## Supplementary Figure S10

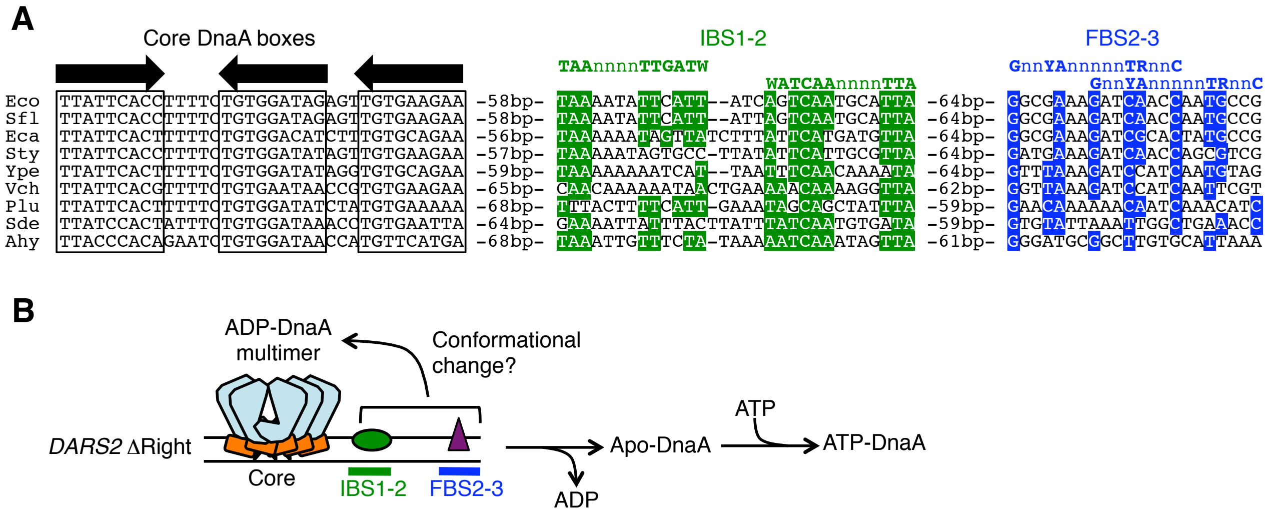

Supplement: SUPPLEMENTARY DATA [file supp_gku1051_nar-01574-m-2014-File009.pdf]
